# In skeletal muscle and neural crest cells, SMCHD1 regulates biological pathways relevant for Bosma syndrome and facioscapulohumeral dystrophy phenotype

**DOI:** 10.1093/nar/gkad523

**Published:** 2023-06-19

**Authors:** Camille Laberthonnière, Mégane Delourme, Raphaël Chevalier, Camille Dion, Benjamin Ganne, David Hirst, Leslie Caron, Pierre Perrin, José Adélaïde, Max Chaffanet, Shifeng Xue, Karine Nguyen, Bruno Reversade, Jérôme Déjardin, Anaïs Baudot, Jérôme D Robin, Frédérique Magdinier

**Affiliations:** Aix Marseille Univ, INSERM, Marseille Medical Genetics, Marseille 13005, France; Aix Marseille Univ, INSERM, Marseille Medical Genetics, Marseille 13005, France; Aix Marseille Univ, INSERM, Marseille Medical Genetics, Marseille 13005, France; Aix Marseille Univ, INSERM, Marseille Medical Genetics, Marseille 13005, France; Aix Marseille Univ, INSERM, Marseille Medical Genetics, Marseille 13005, France; Aix Marseille Univ, INSERM, Marseille Medical Genetics, Marseille 13005, France; Aix Marseille Univ, INSERM, Marseille Medical Genetics, Marseille 13005, France; Aix Marseille Univ, INSERM, Marseille Medical Genetics, Marseille 13005, France; Aix Marseille Univ, INSERM, CNRS, Institut Paoli Calmette, Centre de Recherche en Cancérologie de Marseille, Laboratory of predictive Oncology, Marseille 13009, France; Aix Marseille Univ, INSERM, CNRS, Institut Paoli Calmette, Centre de Recherche en Cancérologie de Marseille, Laboratory of predictive Oncology, Marseille 13009, France; Department of Biological Sciences, National University of Singapore, Singapore 117558, Singapore; Genome Institute of Singapore, A*STAR, Singapore, Singapore; Aix Marseille Univ, INSERM, Marseille Medical Genetics, Marseille 13005, France; Département de Génétique Médicale, AP-HM, Hôpital d’enfants de la Timone, Marseille 13005, France; Genome Institute of Singapore, A*STAR, Singapore, Singapore; Department of Medical Genetics, Koç University, School of Medicine, Istanbul, Turkey; Department of Physiology, Cardiovascular Disease Translational Research Programme, Yong Loo Lin School of Medicine, National University of Singapore, Singapore; Laboratory of Human Genetics & Therapeutics, Smart-Health Initiative, BESE, KAUST, Thuwal, Saudi Arabia; Institut de Génétique Humaine, UMR 9002, CNRS–Université de Montpellier, Montpellier 34000, France; Aix Marseille Univ, INSERM, Marseille Medical Genetics, Marseille 13005, France; Aix Marseille Univ, INSERM, Marseille Medical Genetics, Marseille 13005, France; Aix Marseille Univ, INSERM, Marseille Medical Genetics, Marseille 13005, France

## Abstract

Many genetic syndromes are linked to mutations in genes encoding factors that guide chromatin organization. Among them, several distinct rare genetic diseases are linked to mutations in *SMCHD1* that encodes the structural maintenance of chromosomes flexible hinge domain containing 1 chromatin-associated factor. In humans, its function as well as the impact of its mutations remains poorly defined. To fill this gap, we determined the episignature associated with heterozygous *SMCHD1* variants in primary cells and cell lineages derived from induced pluripotent stem cells for Bosma arhinia and microphthalmia syndrome (BAMS) and type 2 facioscapulohumeral dystrophy (FSHD2). In human tissues, SMCHD1 regulates the distribution of methylated CpGs, H3K27 trimethylation and CTCF at repressed chromatin but also at euchromatin. Based on the exploration of tissues affected either in FSHD or in BAMS, i.e. skeletal muscle fibers and neural crest stem cells, respectively, our results emphasize multiple functions for SMCHD1, in chromatin compaction, chromatin insulation and gene regulation with variable targets or phenotypical outcomes. We concluded that in rare genetic diseases, *SMCHD1* variants impact gene expression in two ways: (i) by changing the chromatin context at a number of euchromatin loci or (ii) by directly regulating some loci encoding master transcription factors required for cell fate determination and tissue differentiation.

## Graphical Abstract

**Graphical Abstract:**
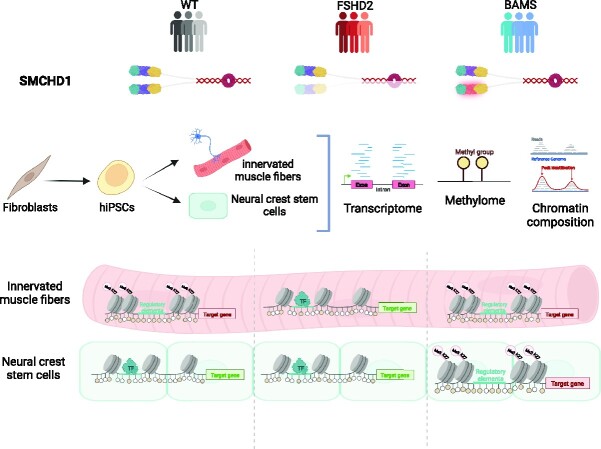

## INTRODUCTION

Based on the presence of an SMC hinge domain, the SMCHD1 (structural maintenance of chromosomes flexible hinge domain containing 1) chromatin-associated factor belongs to the SMC family of chromosomal proteins (1). However, although it forms homodimers by interacting through its SMC hinge domains, SMCHD1 does not participate in the tripartite ring complex formed by other cohesins (2). In mice, Smchd1 is mainly implicated in X inactivation and its depletion causes embryonic lethality with a derepression of genes on the inactive X (Xi) chromosome (3–5). The protein is also involved in the topological conformation of the Xi (6–8) and A/B mega-compartments (6,9–11). Smchd1 plays a role in the regulation of heterochromatin, repetitive DNA sequences or clustered imprinted genes and monoallelically expressed protocadherins (1,3,5,7,12–17). In addition, SMCHD1 binds to telomeres with a direct correlation between telomere length and SMCHD1 enrichment (18,19). However, its precise mechanism of action at telomeres remains partially understood.

Heterozygous germline *SMCHD1* mutations have been reported in at least three distinct rare human genetic diseases, type 2 facioscapulohumeral muscular dystrophy (FSHD2, OMIM #158901) (20–22), Bosma arhinia and microphtalmia syndrome (BAMS, OMIM #603457) (23,24) and isolated hypogonadotrophic hypogonadism with combined pituitary hormone deficiency and septo-optic dysplasia (25). In addition, this gene is often deleted in patients carrying an 18p deletion and presenting symptoms that differ from the three above-mentioned diseases (26,27) or some displaying a typical FSHD phenotype (28). In BAMS, mutations are clustered within exons 3–13, spanning the GHKL-type ATPase domain and an associated region immediately C-terminal to it, while in FSHD2 mutations are dispersed across the whole coding sequence (23,24). In BAMS, many mutations have been associated with a gain of the SMCHD1 ATPase activity (gain-of-function mutation) (23,24,29), while in FSHD2 missense, splice and truncating mutations lead to a loss of function or haploinsufficiency (21,23,29,30).

FSHD is linked in 95% of cases (FSHD1, OMIM #158900) to the shortening of an array of GC-rich 3.3-kb macrosatellite elements (D4Z4) in the 4q arm subtelomeric region (31,32). A smaller proportion of patients (2–3%) carry a mutation in *SMCHD1* (FSHD2) (21). D4Z4 DNA hypomethylation is a cardinal feature of FSHD (20,33–37), but the macrosatellite is also hypomethylated to the same level in BAMS, as well as in 18p11.32 hemizygous patients (23,24,30). Thus, *SMCHD1* haploinsufficiency and loss- or gain-of-function mutations are all associated with D4Z4 hypomethylation with various phenotypical outcomes.

In animal models, *Smchd1* invalidation does not recapitulate the BAMS or FSHD phenotype (23,24). In human tissues, *SMCHD1* heterozygous mutations are not associated with the massive defects in X inactivation observed in mice (30). Thus, how the different heterozygous mutations lead to these diseases affecting the muscle (FSHD) or formation of the nasal placode, migration of surrounding neural crest cells and projection of the gonadotrophin-releasing hormone neurons (BAMS) must be established together with the identification of biological processes (BPs) that depend on SMCHD1.

To tackle this question of the role of SMCHD1 in human tissues, we took advantage of primary cells and induced pluripotent stem cells (hiPSCs) derived from patients affected either with BAMS or with FSHD2. We showed that *SMCHD1* heterozygous mutations impact two major epigenetic determinants, DNA methylation (DNAme) and chromatin architecture, with distinct phenotypical outcomes depending on the tissue. Given SMCHD1 potential implication in the regulation of genes entailed for development and tissue differentiation, we further propose that variegated gene expression triggered by *SMCHD1* variants might contribute to the clinical spectrum of ‘SMCHDnopathies’.

## MATERIALS AND METHODS

### Samples

All individuals had provided written informed consent. The study was done in accordance with the Declaration of Helsinki. Controls were neither carrier of any genetic mutation nor affected by any constitutive pathology and were randomly chosen in the age range and sex representation of patients. All iPSC clones were derived from primary fibroblasts (Supplementary Table S1) (38,39). hiPSCs were treated as described for neuromuscular (40,41) or neural crest stem cell (NCSC) (42) differentiation (Supplementary Table S1).

### Cell culture

The human embryonic kidney 293 (HEK293) cell line (CRL-1573) was obtained from ATCC. In HEK293 cells, *SMCHD1* expression was invalidated by transfection of zinc finger nucleases. Cells were provided by J. Déjardin. Primary fibroblasts and HEK293 cells were grown in Dulbecco’s modified Eagle’s medium (DMEM) with l-alanyl-l-glutamine (GlutaMAX™-I), d-glucose and sodium pyruvate (Life Technologies). Media were supplemented with 10% fetal bovine serum (FBS, Gibco) and 1% penicillin/streptomycin. Cells were grown at 37°C, 5% CO_2_, in a humidified atmosphere. The hiPSCs were differentiated into functional muscles according to the protocol described in (40,41). For chromatin immunoprecipitation sequencing (ChIP-seq) and RNA sequencing (RNA-seq) experiments, cells were collected 30 days post-differentiation. Neural crest differentiation was described in (42). Cells were collected at day 11.

### Transfections

Transfections were performed with 250 mM CaCl_2_ and 2× BBS (50 mM BES, 1.5 mM Na_2_HPO_4_, 280 mM NaCl). Cells were collected 48–72 h post-transfection. For *SMCHD1* knockdown, 1.5 × 10^5^ cells were cultivated in six-well plates. At 70–80% confluence, we replaced the medium with 500 μl of DMEM + 10% FBS + 0.3 μl of polybrene (6 μg/ml, final concentration). Cells were transduced with 6 μl of pLKO.1-puro-CMV-tGFP (Sigma–Aldrich, St Louis, MO; ref: TRCN0000253776) and 6 μl of pLKO.1-puro-CMV-tGFP (Sigma–Aldrich, St Louis, MO; ref: TRCN0000253778). As scramble, we used 12 μl of MISSION^®^ TRC2 Control Transduction Particle puro TurboGFP (Sigma–Aldrich, St Louis, MO; ref: TRCN0000253776) at a multiplicity of infection of 1. Puromycin (Sigma–Aldrich, St Louis, MO; ref: P8833-25MG) was added 24 h after transduction at a final concentration of 1 μg/ml. Clones were harvested after 15 days of culture for RNA, DNA and protein extraction.

### RNA extraction, quality control and library preparation

For high-throughput RNA-seq, total RNA was extracted using the RNeasy kit (Qiagen) following manufacturer’s instructions. Quality, quantification and sizing of total RNA were evaluated using the RNA 6000 Pico assay (Agilent Technologies; ref: 5067-1513) on an Agilent 2100 Bioanalyzer system. The RNA integrity number (RIN) was calculated for each sample and only samples with a RIN > 9 were used. RNA-seq libraries were generated from 600 ng of total RNA using TruSeq Stranded mRNA Library Prep Kit and TruSeq RNA Single Indexes kits A and B (Illumina, San Diego, CA), according to manufacturer’s instructions. The final cDNA libraries were checked for quality and quantified using capillary electrophoresis. Libraries were sequenced at the IGBMC GenomeEast facility using an Illumina HiSeq 4000 1 × 50 bp. For quantitative polymerase chain reaction (PCR) analysis, total RNA was extracted using TRIzol (Thermo Fisher).

### Quantitative reverse transcriptase PCR

Reverse transcription of 1 μg of total RNA was performed using the Superscript IV First-Strand cDNA Synthesis Kit (Life Technologies) using a mix of oligo dT and random hexamers following manufacturer’s instructions. Primers were designed using Primer Blast (Supplementary Table S2). Real-time PCR amplification was performed on a QuantStudio 5 Real-Time PCR System (Thermo Fisher) using the SYBR Green Master Mix (Roche). All PCRs were performed using a standardized protocol and data were analyzed with the QuantStudio 5 Real-Time PCR System (Thermo Fisher). For each sample, fold change (FC) was obtained by comparative quantification and normalization to expression of the *GAPDHD*, *HPRT* and *PPIA* housekeeping genes used as standard. Data are expressed as mean ± standard deviation (SD).

### DNA extraction and sodium bisulfite sequencing

DNA was extracted from the different types of samples using the NucleoSpin Tissue (Macherey-Nagel) according to manufacturer’s instructions.

For sodium bisulfite sequencing, 1 μg of genomic DNA was denatured for 30 min at 37°C in NaOH 0.4N and incubated overnight in a solution of 3 M sodium bisulfite (pH 5) and 10 mM hydroquinone as previously described (43). Primers were designed in order to amplify methylated and unmethylated DNA with the same efficiency using the MethPrimer software avoiding the presence of CpGs in the primer sequence (Supplementary Table S3). After sequencing, sense and antisense sequences were assembled in a single sequence and BAM file converted to fastq file. After trimming of each BSP primer, data were aligned using the BiQ Analyzer HiMod software and processed in R as described in (44). Three methylation scores were calculated: (i) the CpG methylation score of each CpG, (ii) the sequence methylation score, corresponding to the average methylation level of each sequence, and (iii) the global methylation score that corresponds to the global level of methylation for each biological sample calculated as the ratio of methylated CpG with the number of aligned CpGs for all sequences and CpG.

### Luciferase assays

The different experimental DNA fragments were synthetized by GenScript or after PCR amplification and inserted into pGL3 luciferase reporter vectors (pGL3 enhancer and pGL3 promoter, Promega) at the polylinker NheI (NEB) restriction site. Extraction and purification were achieved using NucleoBond^®^ Xtra Midi/Maxi Kit (Macherey-Nagel). Orientation and integrity of the insert were verified by Sanger sequencing. For each experiment, pGL3 firefly luciferase experimental vector has been cotransfected with the pGL4.74[hRluc/TK] Renilla luciferase plasmid used as transfection control. Plasmids were mixed in a 10:1 proportion. For transfection, cells were plated 24 h before transfection. Luminescence was measured using the Dual-Glo^®^ Luciferase Assay System (Promega) kit in a GloMax^®^ Explorer Multimode Microplate Reader. Solutions were dispensed through automatic injectors with 2 s delay and 10 s measurement for each reporter. Experiments were realized in triplicates and measures were performed in triplicates for each construct (*n* = 9). Values were normalized to Renilla luciferase levels. Positions of constructs are described in Supplementary Table S4.

### Position effect variegation assays

The pCMV-derived plasmid is described in (45). DNA inserts were obtained from GenScript and inserted at the AscI restriction site downstream of the eGFP reporter gene in pCMV vectors. Details are available upon request. Transfection of the linearized vectors was performed using a modified calcium phosphate method optimized in order to obtain a single integration per cell (46). Three days post-transfection, the hygromycin B selection antibiotic was added to the culture medium (Life Technologies) at a final concentration of 400 μg/ml. Cells were kept under permanent selection for several passages. At different time points, eGFP expression was analyzed using an Accuri flow cytometer and processed using the FlowJo software (Becton-Dickinson). The percentage of eGFP-positive cells was determined using the corresponding nontransfected cells as the baseline for autofluorescence. Mean values were used to compare fluorescence in the different samples.

### RNA-seq data processing and differential expression analysis

RNA-seq libraries were generated from 600 ng of total RNA using TruSeq Stranded mRNA Library Prep Kit and TruSeq RNA Single Indexes kits A and B (Illumina, San Diego, CA) from four different FSHD2 samples, three BAMS samples and two controls, according to manufacturer’s instructions. Fastq sequence data quality was assessed at the IGBMC facility using FastQC v0.11.5 and reads were trimmed to remove adapter sequences and low‐quality bases using DimerRemover v0.9.2. Trimmed single‐end reads were aligned using STAR v2.5.3a (6) to the hg38 human genome release. BAM files were sorted and indexed using Sambamba (v0.6.6). Stringtie (v1.3.1c) was used to quantify aligned reads with GENCODE. Differentially expressed genes (DEGs) were identified using the DESeq2 (v1.18.1) R package. Heatmaps were built using the R package pheatmap (v1.0.12) and TPM counts. Overrepresentation enrichment analyses were conducted using the clusterProfiler (v3.14.3) R package or Panther, using DEGs with a false discovery rate (FDR)-adjusted *P*-value <0.05 and abs(log_2_FC) > 2 as input.

### Infinium MethylationEPIC array

Genome-wide DNAme analysis was performed by Infinium MethylationEPIC array through Diagenode services. Genomic DNA was extracted using the NucleoSpin Tissue Kit (Macherey-Nagel) from two different cell pellets for each sample (BAMS, *n* = 3; FSHD2, *n* = 3; controls, *n* = 2). The analysis was mainly carried out using the ChAMP R package (47). Probes were filtered out if they had missing values, had a detection *P*-value >0.01 in at least one sample or had a bead count <3 in at least 5% of samples. Samples with >10% of probes with a detection *P*-value >0.01 were filtered out. An annotation file that contains information about the location of probes (chromosome, position and nearby genes) (48) was loaded. Probes targeting CpG sites near single-nucleotide polymorphisms, that belong to X/Y chromosomes or that align with multiple locations were filtered out. For each probe, the methylation level of CpGs covered by the array was determined with 99% confidence by calculating the median DNAme *β*-values and SD within sample groups. Beta values indicate the percentage of CpG sites from a given sample that are methylated (49). Beta values are determined for each CpG location as the relative intensity of methylated and unmethylated signals (50). Probes with an absolute difference (mean Δ*β*-values) of 0.2 and an adjusted *P*-value <0.05 between sample groups were considered as differentially methylated (50). The ChAMP function for identifying differentially methylated probe (DMPs) uses the limma package (51). An ordinary least squares linear model is fit to the methylation values of each probe to estimate the effect of belonging to a phenotype versus control group. Moderated *t*-statistics are used to calculate *P*-values, and the *P*-values are adjusted with the Benjamini–Hochberg method. The Probe Lasso method was used to identify differentially methylated regions (DMRs) using the champ.DMR function. Probe Lasso selects DMPs (default cutoff *P*-value = 0.05) and extends a lasso in either direction. The size of the lasso depends on the gene feature [e.g. body, intergenomic region (IGR)] and CGI relation (e.g. island, shore) of probes. Mini-DMRs are created when the number of DMPs contained within a lasso is greater than or equal to a minimum value. Overlapping or neighboring mini-DMRs are merged until the distance between merged DMRs is at least 1000 bp. A *P*-value is calculated for each DMR, using Stouffer’s weighted method to combine the *P*-values of the probes contained in the DMR. The default cutoff *P*-value for DMRs to be selected is 0.05, and they must contain at least seven probes and exceed 50 bp in width. The Combat method was used to correct for batch effects (52). Heatmaps were built using the pheatmap (v1.0.12) R package using the *β*-value matrix for methylation. Violin plots were realized by converting *β*-values to *M*-value (for better visualization) using the lumi (v2.42.0) R package and then by plotting values using the ggplot2 (v3.3.3) R package. DMPs with an FDR-adjusted *P*-value <0.05 and abs(Δ*β*) > 0.2 were represented as bar plots using the ggplot2 (v3.3.3) R package for genomic features and CGI status according to Illumina Epic array annotation file. Karyoplots of DMPs with an FDR-adjusted *P*-value <0.05 and abs(Δ*β*) > 0.2 were realized using the KaryoplteR (v1.16.0) R package. DMPs were represented as a density measured in bins. For karyoplots, DMPs with FDR < 0.05 and abs(Δ*β*) > 0.5 were plotted as dot over chromosomes with the *y*-axis ranging from 0 to 1.

### ChIP and ChIP-seq data processing

Cross-linking of 10–20 × 10^6^ cells was performed as described (38). We used the iDeal ChIP-seq Kit for Transcription Factors and iDeal ChIP-seq Kit for Histones (Diagenode s.a., Seraing, Belgium), as recommended by the manufacturer in hiPSC-derived muscle fibers (MFs) and NCSCs for two FSHD2, two BAMS and controls. For ChIP-qPCR, 5 μg of antibodies against SMCHD1 (Abcam ab31865 and Sigma HPA039441) were mixed. Primers are described in Supplementary Table S5. ChIP-grade antibodies against CTCF, H3K4me3, H3K27Ac or H3K27me3 were used as recommended (Diagenode). ChIP samples were purified using Agencourt AMPure XP beads (Beckman Coulter) and quantified with the Qubit (Invitrogen). ChIP-seq libraries were prepared from 10 ng of double-stranded purified DNA using the MicroPlex Library Preparation Kit v2 (C05010014, Diagenode), according to manufacturer’s instructions. Illumina compatible indexes were added through a PCR amplification (seven cycles). Amplified libraries were purified and size selected using Agencourt AMPure XP beads (Beckman Coulter) to remove unincorporated primers and other reagents. Libraries were sequenced at the IGBMC GenomEast facility using an Illumina HiSeq 4000 1 × 50 bp. Sequence reads were mapped to reference genome hg38 using Bowtie 1.0.0 with the following parameters: -m 1 –strata –best -y -S -l 40 -p 2 and delivered as BAM files along with Wig files generated using an in-house script at IGBMC (variable step, span = 25, reads were elongated to 200 bp). BAM files were analyzed using MACS2 (v2.2.6) using input DNA as control. Peaks were identified using broad peak calling for H3K4me3, H3K27Ac and H3K27me3 and sharp peak calling for CTCF, both with a *q*-value threshold <0.05. For samples with lower chip signals, we used no model parameter with extsize = 200 bp. Peaks’ correspondence between replicates was assessed using MSPC to obtain consensus peaks. Using –B parameter, bedGraph files were generated and whenever possible the replicates were merged and then converted to bigWig files for data visualization. Karyoplots of bigWig files were realized using the KaryoplteR (v1.16.0) R package. Tracks are scaled to the highest value found in the window for each mark. Overrepresentation was determined using the clusterProfiler (v3.14.3) R package. Genes overlapping identified peaks with at least a *q*-value <0.05 in one replicate were used as input. Identified Gene Ontology (GO) terms in biological process (BP) ontology were selected based on an FDR-adjusted *P*-value <0.05. Intersection of genomic coordinates from ChIP-seq peaks was realized using Intervene (18).

We also analyzed a publicly available data set for SMCHD1 distribution in HCT116 cells (https://www.ncbi.nlm.nih.gov/geo/query/acc.cgi?acc=GSM1130654). Sites with SMCHD1 enrichment were determined by peak calling and comparison between HCT116 cells and knockout (KO) cells. A .bed genomic coordinate file containing the coordinates of 526 peaks of SMCHD1 from a MACS2 peak calling analysis was used. From this coordinate file, a fasta .fa sequence file using bedtools getfasta was created.

## RESULTS

### SMCHD1 deficiency results in both increased and decreased DNAme at the genome-wide level

**Figure 1.**
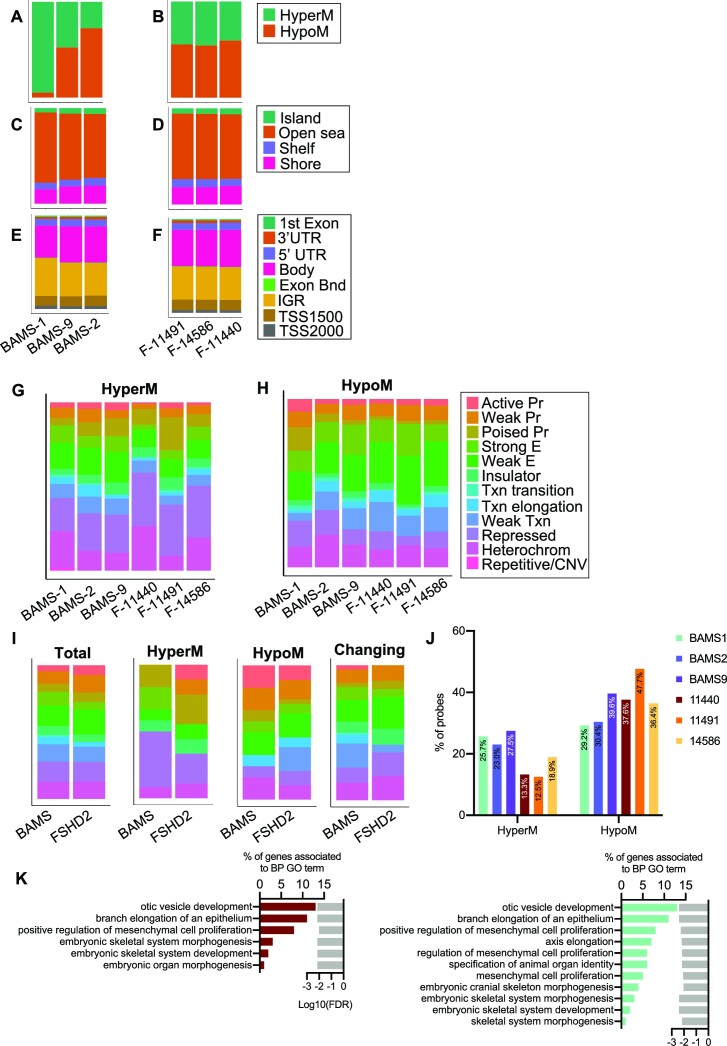
Profiling of DNAme in SMCHD1-deficient cells. One hundred percent stacked bar graphs of the distribution of hypomethylated and hypermethylated probes in individual BAMS samples (**A**) or individual FSHD2 samples (**B**). One hundred percent stacked bar graphs for DMPs by CpG content relative to CpG islands, shores (2 kb flanking CpG islands), shelves (2 kb extending from shores) or open seas (isolated CpGs in the rest of the genome) in individual BAMS samples (**C**) or individual FSHD2 samples (**D**). One hundred percent stacked bar graphs for DMPs by features corresponding to genes’ first exon, 3′ UTR, 5′ UTR, gene bodies, exon boundaries, internal genomic regions and probes located 1500 bp from transcription start sites (TSS1500) or 2000 bp from transcription start sites (TSS200) in individual BAMS samples (**E**) or individual FSHD2 samples (**F**). DMRs with an FDR-adjusted *P*-value <0.05 were analyzed for ChromHMM features using NHEK cell annotations and plotted using the ggplot2 (v3.3.3) R package. One hundred percent stacked bar graphs for hypermethylated (**G**) or hypomethylated (**H**) probes are presented. (**I**) Left to right: bar plots of DMRs shared between patients when compared to controls (total), shared hypermethylated DMRs (HyperM), shared hypomethylated DMRs (HypoM) and DMRs with a different methylation profile between patients (changing). (**J**) Graph displaying the percentage of hypermethylated or hypomethylated probes at enhancers in the different BAMS and FSHD2 samples. (**K**) BPs overrepresented for BAMS (cyan, right) or FSHD2 (red, left) DMRs. Bar plots on the left represent the percentage of genes and associated with GO terms listed in the right column. Light gray bars on the right represent the enrichment score (log_10_FDR) for each GO term.

Considering the role of SMCHD1 in DNAme (21,23,24,30), we first asked whether genome-wide methylation profiles might distinguish BAMS from FSHD2 and control cells. To address this question, we performed DNAme profiling in primary fibroblasts at early passages (<10 passages; BAMS, *n* = 3; FSHD2, *n* = 3; controls, *n* = 2; Supplementary Table S1) after sodium bisulfite conversion using the Illumina Infinium HumanMethylation 850 BeadChip (53). Density plot analyses and unsupervised hierarchical clustering indicated an overall overlapping of profiles between samples (Supplementary Figure S1A–D). We identified 29 528 DMPs in BAMS (3% of the probes) and 53 709 DMPs in FSHD2 (6% of the probes), including 12 054 DMPs shared between the two diseases (Supplementary Table S6 and Supplementary File S1). Compared to controls, methylation is globally increased in BAMS cells but decreased in FSHD2 (20% of disease-associated DMPs; Supplementary Figure S1E). Changes occur across all autosomes (Supplementary Figure S2). However, the differences between the two diseases might be explained in part by the discrepancy in the proportion of hypermethylated probes in the different BAMS samples, with a higher proportion of hypermethylated probes in BAMS1, compared to the balanced proportion of hypermethylated and hypomethylated probes in all FSHD2 samples (Figure 1A and B, Supplementary Figure S1E and Supplementary Table S6).

In the two diseases, the distribution of DMPs by CpG content categorized using the Illumina annotations is equivalent. The majority of DMPs correspond to open seas (CpGs outside CpG islands) (Figure 1C and D, Supplementary Figure S1F and Supplementary Table S6), gene bodies and IGRs (Figure 1E and F, Supplementary Figure S1G and Supplementary Table S6). A large proportion of DMPs map to the 6p21.32–6p22.1 locus encompassing the major histocompatibility complex locus and genes encoding chromatin factors and microRNAs (Supplementary Figure S3A).

Next, we searched for DMRs by using the Probe Lasso method (48). When compared to controls, we found between 70 and 263 DMRs in BAMS samples and between 49 and 110 DMRs in FSHD2 samples, distributed across all autosomes (Supplementary Figures S2A and B and S3B–E, and Supplementary File S1). We then selected DMRs shared between BAMS and FSHD2 and sorted hypermethylated and hypomethylated DMRs or DMRs with an opposite methylation status (Supplementary Tables S7 and S8, and Supplementary Figure S3B–E). For prediction of functional elements showing DNAme changes, we analyzed them using the ChromHMM track from NHEK (normal epidermal keratinocytes, Broad Institute; Encode). NHEKs were used as the closest tissue type relative to the patient’s arm dermal primary fibroblasts used in our analyses and the tissue with the closest anatomical location. The largest proportion of DMRs corresponds to repressed chromatin/heterochromatin or repressed and poised promoters (Figure 1G–I, and Supplementary Tables S7 and S8). Interestingly, we also noticed a marked hypomethylation at enhancers in patient’s cells with 29.23–39.64% of hypomethylated DMRs at enhancers in BAMS and 36.42–47.66% in FSHD2 (Figure 1J, and Supplementary Tables S7 and S8). GO BPs associated with genes in the vicinity of these hypomethylated enhancers encode factors involved in developmental processes (Figure 1K and Supplementary File S1), including genes related to skeletal muscle system development and cell differentiation. Comparisons were also made with adult primary lung fibroblasts and yield comparable results (not shown).

Overall, the high proportion of hypomethylated probes in cells carrying a variant in *SMCHD1* confirms a role for this factor in directing DNAme. On the other hand, increased DNAme at heterochromatin might also suggest that shielding against deposition or spreading of this epigenetic mark might be impaired in patient’s cells. Changes of methylation at enhancers in *SMCHD1*-mutated cells further suggest a role for SMCHD1 in the regulation of *cis*-regulatory elements in human tissues.

### In pluripotent cells, *SMCHD1* variants impact methylation of a small subset of DNA sequences

After cell reprogramming, methylation profiles overlap between controls and SMCHD1-deficient cells (Supplementary Figure S4A and B, and Supplementary Table S9). Compared to fibroblasts, we observed an opposite distribution in DNAme between the two diseases, with a majority of hypomethylated probes in BAMS but a majority of hypermethylated probes in FSHD2 (Supplementary Figure S4C). As in fibroblasts, most DMPs are located in gene bodies and IGRs (Supplementary Figure S4E), with only 211 DMPs shared between the two diseases (Supplementary Figure S4F). Besides gene bodies and IGRs, we also observed a higher proportion of DMPs in CpG islands in BAMS and a higher proportion in open seas and shelves for FSHD2 hiPSCs (Supplementary Figure S4D). These results indicate a massive reset of the DNAme profile after cell reprogramming with distinct genome-wide impacts depending on the type of *SMCHD1* variants.

### Mutations in *SMCHD1* are associated with changes in the expression of a small subset of protein-coding genes

**Figure 2.**
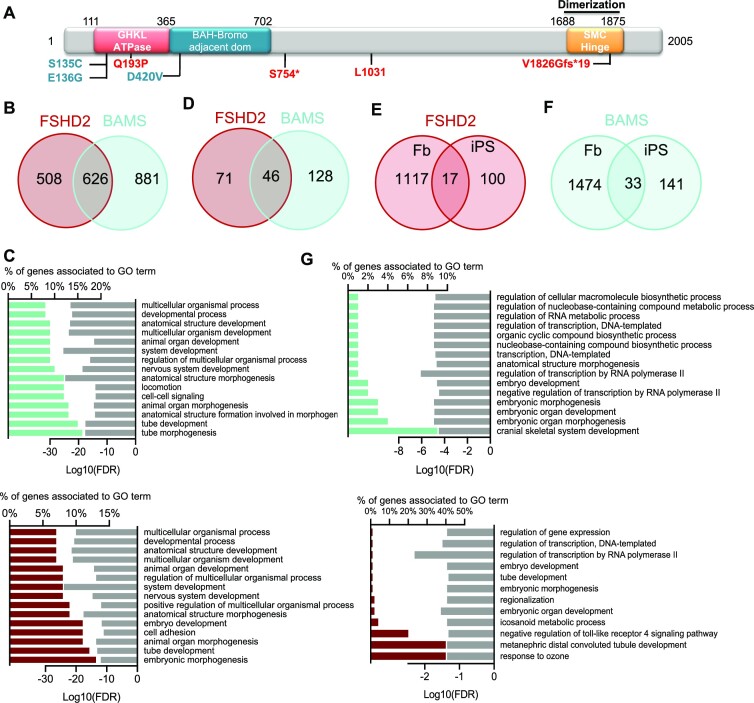
Expression profiling in fibroblasts and iPSCs from patients affected with BAMS or FSHD2. (**A**) Schematic representation of the SMCHD1 protein with position of the different mutations in BAMS (cyan) or FSHD2 (red) patients. (**B**) Venn diagrams for comparison of genes that are differentially expressed in FSHD2 and BAMS primary fibroblasts compared to controls with −2 > FC > 2 and FDR < 0.05. (**C**) GO for BPs corresponding to enrichment analysis of DEGs in FSHD2 (red, upper panel) or BAMS (cyan, lower panel) versus control fibroblasts filtered on −2 > FC > 2 and FDR < 0.05. Bar plots on the left represent the percentage of genes that are differentially expressed and associated with a GO term shown in the right column. Light gray bars on the right represent the enrichment score (log_10_FDR) for each BP. (**D**) Venn diagrams for comparison of DEGs in FSHD2 and BAMS hiPSCs compared to controls. (**E**) Venn diagrams for comparison of DEGs between FSHD2 fibroblasts and hiPSCs. (**F**) Venn diagrams for comparison of DEGs between BAMS fibroblasts and hiPSCs. (**G**) GO for BPs corresponding to enrichment analysis of DEGs in FSHD2 (red, upper panel) or BAMS (cyan, lower panel) versus control hiPSCs filtered on −2 > FC > 2 and FDR < 0.05. Bar plots on the left represent the percentage of DEGs and associated with GO terms in the right column. Light gray bars on the right represent the enrichment score (log_10_FDR) for each GO term.

In order to test whether SMCHD1 variants alter gene expression, we searched for genes dysregulated in primary fibroblasts from the same three BAMS and three FSHD2 patients carrying variants in *SMCHD1* (Figure 2A). In comparison to healthy donors (controls), we identified 1134 DEGs in FSHD2 cells and 1507 in BAMS cells, with an overlap of 626 DEGs between the two diseases (FC ≥ 2; *P*-value <0.05; Figure 2B, Supplementary Figure S5A and B, and Supplementary File S2). Only a few genes show an opposite trend of expression (up in BAMS and down in FSHD2: *KRT7*, *HAPLN1* or vice versa, *EMB*, *MEOX2* and *LCNL1*). The analysis of BPs for BAMS and FSHD2 DEGs revealed that 11 out of the 15 most significant processes are related to development and body patterning and shared between the two diseases (Figure 2C).

In hiPSCs, we identified a smaller number of DEGs (117 in FSHD2 and 174 in BAMS), with an overlap of 46 DEGs between the two diseases (FC ≥ 2, *P*-value <0.05; Figure 2D, Supplementary Figure S5C and D, and Supplementary File S2). After reprogramming, the expression of ∼90% of the DEGs found in fibroblasts is thus reset with only 17 DEGs shared between primary fibroblasts and hiPSCs for FSHD2 (Figure 2E) and 33 genes in BAMS (Figure 2F). Associated BPs correspond to various cellular functions or developmental processes with 3 out of 15 of the most significant pathways common between the two diseases (Figure 2G).

SMCHD1 loss of function, D4Z4 chromatin relaxation and subsequent *DUX4* activation are proposed as FSHD driver mechanisms. In fibroblasts, *DUX4* is undetectable by RNA-seq due to its low expression level. Only a small number of the 422 DUX4 target genes (54) are among DEGs, i.e. 19 genes in BAMS and 11 in FSHD2, including 5 common to both diseases (Supplementary Figure S5E). In hiPSCs, only one DUX4 target (*OAS1*) was identified among DEGs in BAMS (Supplementary Figure S5F).

As described previously, cells carrying *SMCHD1* variants (BAMS or FSHD2) display differential expression of several genes associated with development and cell differentiation but are not distinguishable based on the expression of *DUX4* or of its target genes. As observed for DNAme, gene expression profiles are reset upon reprogramming of primary cells carrying a mutation in *SMCHD1*. Based on this characterization, hiPSCs appear a suitable model for investigating how *SMCHD1* variants might later impact specific tissues during differentiation.

### Absence of correlation between methylation changes and *HOX* gene expression

As a comparison to genes regulated by Smchd1 in KO mice (7), we characterized *HOX* loci more carefully. We analyzed changes in DNAme at the four human *HOX* gene clusters and compared their methylation level to *HOX* gene expression. Methylation profiles are comparable in BAMS and FSHD2 cells. We identified several DMPs across all *HOX* loci (Supplementary Figures S6 and S7, and Supplementary File S1) with a majority of hypermethylated probes for *HOXA*, *HOXB* and *HOXD* clusters and a higher proportion of hypomethylated probes for the *HOXC* locus. Twenty-two *HOX* genes are differentially expressed in BAMS and FSHD2 fibroblasts, but we did not observe any obvious correlation between gene expression and methylation changes (Supplementary Figure S6E and F, and Supplementary Files S1 and S2).

For *HOX* genes, we selected four main DMRs with features of polycomb repressed chromatin (*HOXA13*/*HOTTIP*), poised promoter (*HOXB2*, *HOXB6)* or enhancer (*HOXC4*, *HOXC5*, *HOXC6*) (Supplementary File S1). As not all CGs are covered by the 850k Epic array, we then performed in-depth methylation profiling of these four DMRs by sodium bisulfite sequencing in fibroblasts and hiPSCs. We noted variable DNAme profiles in fibroblasts and hiPSCs. *HOXA13* methylation level is identical between control fibroblasts or hiPSCs and between samples (Supplementary Figure S8A). This DMR is markedly hypomethylated in one of the FSHD2 samples compared to the other FSHD2 and BAMS cells with no apparent correlation with mutations altering SMCHD1 ATPase activity (F-14586, loss of ATPase activity; BAMS1 and BAMS2, gain of ATPase activity). In hiPSCs, the opposite trend is observed, with an increased methylation in cells derived from hypomethylated fibroblasts (F-14586) and an absence of methylation when the DMR is methylated in fibroblasts (F-120521C, BAMS1–BAMS9; Supplementary Figure S8A). The *HOXB2* DMR is unmethylated and remains unmethylated upon reprogramming in the majority of samples (Supplementary Figure S8B). *HOXB6* methylation is low except in BAMS2 fibroblasts [gain of SMCHD1 ATPase activity (29)] but increases after reprogramming in one of the FSHD2 samples [F-14586; loss of ATPase activity (30)] (Supplementary Figure S8C). Interestingly, for *HOXC4*, we observed an absence of methylation in samples carrying a mutation affecting the ATPase activity (F-14586, BAMS1, BAMS2) and an increased methylation in other patient’s fibroblasts (F-120521C, BAMS9). Demethylation of this DMR occurs in all samples after reprogramming (Supplementary Figure S8C). Overall, we observed an absence of correlation between DNAme and gene expression. As in mice, SMCHD1 contributes to the regulation of DNAme profile at autosomal loci in human cells. Nonetheless, as observed for D4Z4 (30), *SMCHD1* germline mutations have different impact on the distribution of methylated CGs.

### *In vitro* or *in vivo* somatic *SMCHD1* invalidation does not modify DNAme

In patient’s cells, SMCHD1 binding to the D4Z4 macrosatellite is DNAme independent (30). D4Z4 hypomethylation is a hallmark of patients carrying a germline *SMCHD1* mutation or heterozygosity of the corresponding locus (23,24,30,36). However, as shown in cancer cell lines (30), somatic *SMCHD1* invalidation in human primary fibroblasts does not cause D4Z4 hypomethylation (Supplementary Figure S9A). To address whether these observations also apply to other genomic sequences, we analyzed DNAme of the four selected *HOX* DMRs (*HOXA13*, *HOXB2*, *HOXB6*, *HOXC4*) in HEK and HEK *SMCHD1* KO cells by sodium bisulfite sequencing. In HEK *SMCHD1* KO cells, somatic *SMCHD1* invalidation and subsequent absence of SMCHD1 binding (Supplementary Figure S9B) does not cause any change in DNAme of *HOX* DMRs (Supplementary Figure S9C). In the absence of SMCHD1, the expression of *HOXB* and *HOXD* genes is variable, while expression of genes in the vicinity of the *HOXA13* and *HOXC4/5/6* DMRs (Supplementary Figure S9D) is increased. As indicated by our analyses in patient’s primary cells, this further highlights a possible role for SMCHD1 in the regulation of *HOX* gene expression, independently of DNA hypomethylation, but also shows that *SMCHD1* KO in primary cells does not directly impact *HOX* gene expression, as observed before (55). As shown here and previously *in vitro* and confirmed here *in vivo* in cancer specimens with a somatic loss of heterozygosity of the 18p locus encompassing *SMCHD1* (Supplementary Figure S10), DNAme changes are only triggered by germline *SMCHD1* mutations. This further confirms a role for SMCHD1 in *de novo* DNAme but not in its maintenance in somatic cells.

### SMCHD1 variants impact the distribution of H3K27me3 during differentiation

Despite the lack of correlation observed between gene expression and DNAme changes, differentially methylated sites are associated with genes that belong to BPs relevant to either BAMS or FSHD2 respective phenotype and associated with development or differentiation.

After characterization of primary cells and hiPSCs from patients carrying a mutation in *SMCHD1*, we then asked how SMCHD1 controls chromatin organization during differentiation. To this aim, we performed chromatin profiling in hiPSC-derived skeletal MFs and NCSCs (42,56) derived from patient’s cells. We mapped the activating H3K4me3, H3K27Ac and repressive H3K27me3 histone marks together with distribution of the CTCF insulating factor by ChIP-seq. Enrichment peaks are distributed along the 22 autosomes for the two diseases in the two cell types (Supplementary Figure S11A and B, and Supplementary Files S3 and S4). Contrarily to DNAme, we did not observe disproportional enrichments at chromosome 6 nor at any other chromosome.

**Figure 3.**
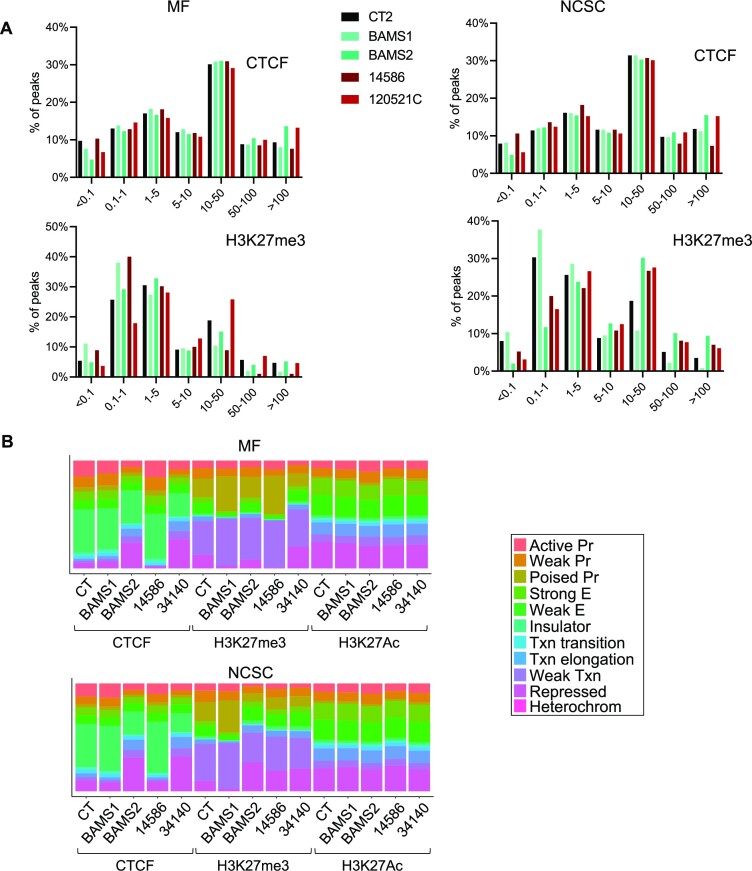
Chromatin profiling of hiPSC-derived MFs and NCSCs from SMCHD1-deficient patients. (**A**) Distribution of distances from TSSs for peaks enriched in CTCF or H3K27me3 in controls, BAMS or FSHD2 MFs or NCSCs. Peaks’ distribution relative to TSSs was assessed using the chipenrich (v2.14.0) R package. (**B**) Distribution of peaks enriched in CTCF, H3K27me3 or H3K27Ac relative to chromatin features determined using the ChromHMM track in MFs or NCSCs derived from control, BAMS or FSHD hiPSCs. Peaks with at least a *q*-value <0.05 in one replicate were analyzed for ChromHMM features using HSMM cell annotations and represented as bar plots using the R ggplot2 (v3.3.3) package.

Relative to transcription start sites (TSSs), CTCF, H3K4me3 and H3K27Ac peaks are evenly distributed in diseased MFs or NCSCs compared to controls (Figure 3A and Supplementary Figure S12A). H3K27me3 distribution is more variable (Figure 3A). The majority of H3K27me3 peaks are located at TSSs (<0.1 kb) or at a short distance (0.1–5 kb) from TSSs. In BAMS and FSHD2 cells, most changes in H3K27me3 enrichment occur at TSSs (0.1 and 0.1–1 kb) but also at a distance of 10–50 kb. In patient’s samples, we observed an opposite trend between these two regions: an increased H3K27me3 enrichment at TSSs (<0.1 kb) corresponds to a decrease at a distance of 10–50 kb from the TSSs and vice versa, suggesting a redistribution of this chromatin mark in patient’s cells.

We then determined chromatin enrichment relative to chromatin features in the different samples using the ChromHMM track for human skeletal muscle myoblast (HSMM) annotation. Consistent with previous analyses, we did not observe major changes for activating chromatin marks (H3K4me3, H3K27Ac) between conditions (Figure 3B, Supplementary Figure S12B, and Supplementary Files S3 and S4). We noticed an increased proportion of CTCF peaks at repressed chromatin and heterochromatin but a decrease at active promoters and insulators. This suggests that SMCHD1 deficiency might be associated with a redistribution of CTCF at heterochromatin to protect against heterochromatin spreading or silencing.

**Figure 4.**
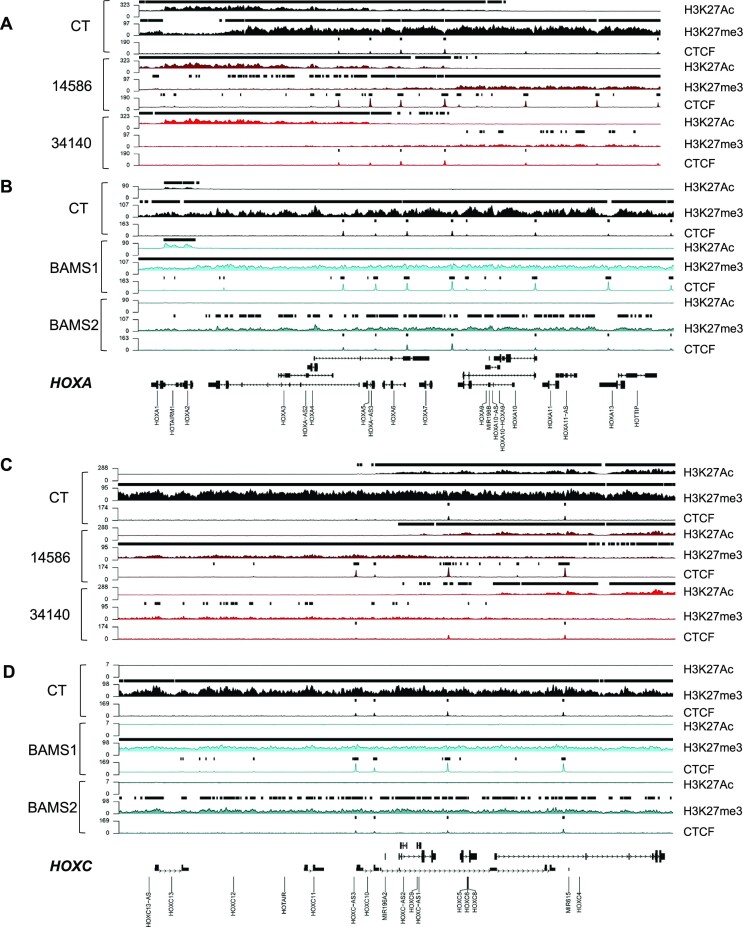
Chromatin profiling of *HOXA* and *HOXC* genes in *SMCHD1*-deficient cells. (**A**) Profiling of the *HOXA* gene locus (chr7:27090339–27211142, 120 803 bp) in controls (black) or FSHD2 (red) MFs for H3K27Ac, H3K27me3 or CTCF enrichment. (**B**) Profiling of the *HOXA* gene locus (chr7:27090339–27211142, 120 803 bp) in controls (black) or BAMS (cyan) NCSCs for H3K27Ac, H3K27me3 or CTCF enrichment. (**C**) Profiling of the *HOXC* gene locus (chr12:53930562–54058000; 127 438 bp) in controls (black) or FSHD2 (red) MFs for H3K27Ac, H3K27me3 or CTCF enrichment. (**D**) Profiling of the *HOXC* gene locus (chr12:53930562–54058000; 127 438 bp) in controls (black) or BAMS (cyan) NCSCs for H3K27Ac, H3K27me3 or CTCF enrichment.

As for DNAme, the H3K27me3 silencing mark is increased at repressed chromatin and poised promoters in several of patient’s samples with differences between proliferative (NCSCs) and post-mitotic differentiated cells (MFs) and type of mutation (gain or loss of the ATPase activity) (Figure 3A). We overall concluded that SMCHD1 deficiency does not lead to a massive loss of repressive chromatin marks (H3K27me3, DNAme), as illustrated at *HOX* loci (Figure 4), but rather to a local redistribution of repressive marks, at promoters, repressed chromatin and heterochromatin. Moreover, based on changes in CTCF distribution, we hypothesized that SMCHD1 might shield against the deposition of repressive chromatin marks and that this shielding might be impaired in SMCHD1-deficient cells, with different repercussions compared to Smchd1 KO mouse cells (7).

### CTCF binding is increased in the total absence of SMCHD1 but not in SMCHD1-deficient cell lineages

As we observed that *SMCHD1* mutations in human cells have a different impact on chromatin features compared to its absence in mouse tissues regarding CTCF binding, we also analyzed publicly available ChIP-seq data (Gene Expression Omnibus, GSM1130654) obtained in a human SMCHD1-null cell line (HCT116). In these cells, *SMCHD1* has been somatically invalidated using a zinc finger nuclease. After filtering for repetitive DNA sequences, we retrieved 526 SMCHD1-enriched peaks mostly located at a distance of 50–500 kb from TSSs. By artificial extension of these SMCHD1 peak coordinates (±10 kb from the peak), we sorted a list of genes in their vicinity and compared this list to the list of fibroblast DEGs (Supplementary File S1). We identified 24 DEGs with 14/24 harboring a CTCF site in their vicinity. To assess whether SMCHD1 might interfere with CTCF enrichment, we selected three loci harboring insulator features and corresponding to three DEGs (*BET1L*, *NCAM2* and *SEMA5A*) together with one gene close to a putative SMCHD1 peak that does not overlap with CTCF (*WASH7PL*; no associated chromatin feature) (Supplementary Table S4). BET1L and WASH7P are located in heterochromatin regions. Sites located close to the *NCAM2* and *SEMA5A* genes correspond to distal enhancers (>50 kb from TSSs). We analyzed CTCF and SMCHD1 enrichment by ChIP-qPCR at all four sites. In HCT116 KO cells compared to HCT116 cells, the absence of SMCHD1 is associated with a significant increase in CTCF occupancy (Supplementary Figure S13, *P*-value <0.00001). We concluded that, as in the mouse (7), the total absence of SMCHD1 impacts CTCF distribution in human cells. Based on these results and those obtained in *SMCHD1*-mutated cells (BAMS and FSHD2 cells), we also concluded that depending on the genomic functional features, SMCHD1 contributes to CTCF distribution in human cells.

### SMCHD1 regulates gene expression *in cis*

Having evidenced a role for SMCHD1 in the distribution of CTCF or H3K27me3, we then tested functionally whether different sequences might regulate gene expression and insulation in an SMCHD1-dependent manner. To this aim, we performed transient and stable reporter gene expression assays in HEK and HEK *SMCHD1* KO cells. Transient reporter assays were used to evaluate enhancer or promoter activity. Stable insertions of single-copy transgenes are used to reveal protection against position effects and insulation (45).

**Figure 5.**
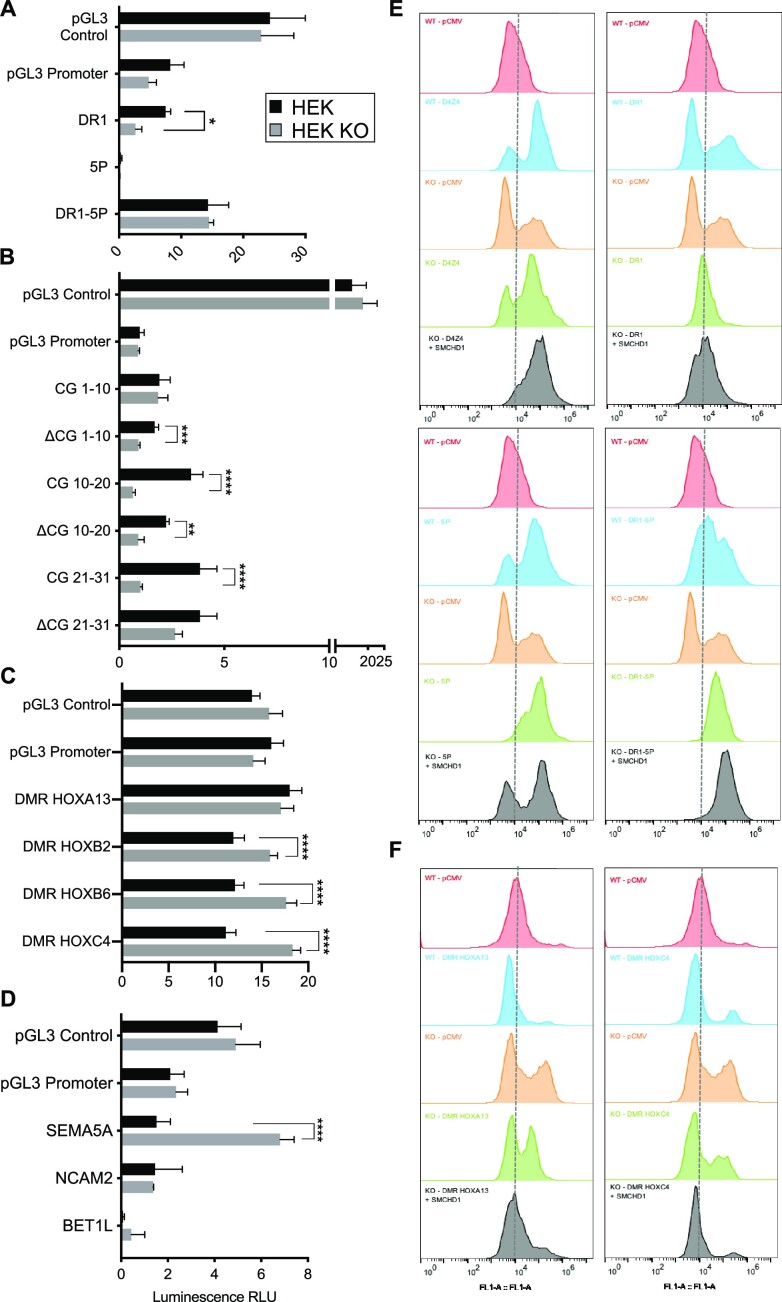
Depending on the genomic context, SMCHD1 contributes to gene silencing or protects against position effects. For the different pGL3 constructs, fragments corresponding to the regions that are differentially methylated (**A**–**C**) or corresponding to SMCHD1 binding sites (**D**) were cloned downstream of the luciferase reporter gene in vectors lacking an enhancer (pGL3 promoter). Firefly luciferase expression was determined 48 h post-transfection of the different constructs in HEK293 and HEK *SMCHD1* KO cells. The pGL3 control vector was used as a transfection control. Firely luciferase levels were normalized to expression of the Renilla luciferase used as transfection control. Values corresponding to the normalized luciferase activity (expressed in relative luminescence units, RLUs) are the average of three independent assays, each realized as technical triplicates (*n* = 9). Error bars represent standard error. Statistical significance was determined using a Mann–Whitney test (****P-value <0.00001, ***P-value <0.0001, **P-value <0.001, *P-value = 0.01). (A) For the D4Z4 macrosatellite, regions that are differentially methylated in patients carrying a mutation in *SMCHD1* were tested (DR1, 5P; a scheme of the D4Z4 repeat is presented in Supplementary Figure S13). (B) The DR1 sequence contains 31 CG sites. Fragments corresponding to CG1–10, CG10–20 and CG21–31 or deleted of 10 of these CGs (ΔCG1–10, ΔCG10–20 and ΔCG21–31) were tested. (C) H*OX* gene DMRs. (D) Putative SMCHD1 binding sites overlapping or not with CTCF binding sites. (**E**, **F**) For evaluation of protection against position effect, we used a vector carrying a hygromycin resistance gene and an *eGFP* reporter gene. Sequences to be tested are cloned downstream of the *eGFP* gene and transfected into HEK or HEK KO cells. Stable eGFP expression was measured by flow cytometry (FACS) for an extended period of time in cells grown in the presence of hygromycin B. Representative spectra of the % of eGFP-positive cells are presented. For each condition, eGFP expression was compared to values obtained in cells transfected with the empty vector (pCMV). In HEK *SMCHD1* KO cells, eGFP expression was also measured 72 h after transfection of an *SMCHD1* expression vector (gray curves). (E) D4Z4 (left upper panel), DR1–5P (right upper panel), DR1 (left lower panel) and 5P (right lower panel). (F) Results obtained for the HOXA13 (left) and HOXC4/5/6 (right) DMRs.

For D4Z4, we selected subregions that are bound by SMCHD1 (30) and that display a differential methylation profile in patients (37,44) on transient luciferase expression (Supplementary Figure S14A). In a vector lacking the SV40 enhancer (pGL3 promoter), the proximal part of D4Z4 differentially methylated in *SMCHD1* KO cells (DR1 and DR1 subfragments, up to CG 21) activates luciferase expression in an SMCHD1-dependent manner (Figure 5A and B). This sequence has no activity in a promoterless reporter (Supplementary Figure S14B). This suggests that the DR1 fragment acts as an SMCHD1-dependent *cis*-activator. For the different *HOX* DMRs bound by SMCHD1 in HEK cells, we did not observe any enhancer activity (Figure 5C). However, in the absence of SMCHD1, luciferase expression increases for *HOXB2*, *HOXB6* and *HOXC4* DMRs (Figure 5C). This shows that SMCHD1 negatively regulates gene expression via these DMRs. We made the same observation for the region near *SEMA5A* identified as a potential SMCHD1 binding site but not for *NCAM2* or *BET1L* (Figure 5D). Using these transient transfection assays, we concluded that SMCHD1 might contribute to both transcriptional activation and silencing depending on its target sequence.

### SMCHD1 protects against position effect variegation in human tissues

Smchd1 has been initially identified as a suppressor of position effect variegation (PEV) (1). PEV is defined as the variable expression of genes due to the stochastic establishment of an epigenetic state modifying their regulatory region. We tested the impact of SMCHD1 on PEV in human cells by monitoring reporter gene expression after stable integration of a single copy of our reporter system (45).

The D4Z4 insulator sequence was first used as a reference (Figure 5E). As indicated by the increased proportion of eGFP-positive cells in D4Z4-containing constructs (Figure 5E, blue shading), D4Z4 protects against PEV in HEK cells. This D4Z4-dependent protection against PEV strongly increases when *SMCHD*1 is re-expressed in HEK KO cells (Figure 5E, gray shading). This shows that SMCHD1 contributes to the protection against PEV mediated by D4Z4. Regarding D4Z4 subfragments, DR1 harbors a moderate anti-PEV that is not improved by *SMCHD1* overexpression. The 5P region located downstream of DR1 or the DR1–5P combination increases the percentage of cells expressing the eGFP reporter. This reveals that protection against PEV is mediated by the proximal part of the D4Z4 repeat and depends on SMCHD1. These results confirm that the D4Z4 proximal part that is hypomethylated in patients displays an SMCHD1-dependent enhancer–insulator activity.

Regarding other loci, the *HOXA13* DMR has no effect on the eGFP reporter expression as in luciferase assays (Figure 5F). By increasing the percentage of eGFP-positive cells, *HOXB2* DMR acts as an activating element partially dependent on SMCHD1 (KO cells, orange shading, Supplementary Figure S14D). The insertion of *HOXB6* (Supplementary Figure S14D) and *HOXC4/C5/C6* (Figure 5F) DMRs slightly increases the percentage of eGFP-positive cells. This percentage is augmented in the absence of SMCHD1 indicating that SMCHD1 negatively regulates *HOXB6* and *HOXC4/C5/C6* DMRs as observed in transient luciferase assays. Regarding the sites identified in HCT116/HCT116 KO cells, similar results were obtained for *NCAM2*, *BET1L* and *WASH7PL* with a marked increase in eGFP-positive cells in HEK KO cells. This marked increase is counterbalanced upon *SMCHD1* re-expression (Supplementary Figure S14D). We concluded that these regions repress gene expression in an SMCHD1-dependent manner, while the effect on gene expression of the site located at the *SEMA5A* locus is not mediated by SMCHD1.

Overall, these results highlight versatile functions for SMCHD1 as a *cis-*activator (D4Z4–DR1, *SEMA5A*), or as a moderate (*HOXB2*, *NCAM2*, *WASH7PL*) to strong (*HOXA13*, *HOXB6*, *HOXC4/C5/C6*, *BET1L*) repressor of gene expression.

### SMCHD1 regulates BPs relevant to BAMS and FSHD2 phenotype in patient’s derived cells

**Figure 6.**
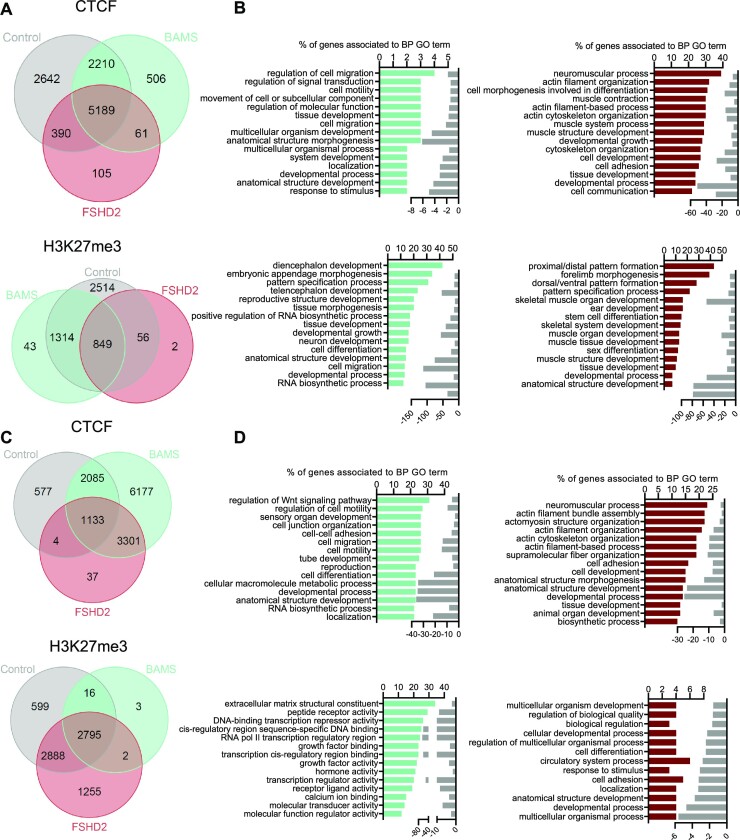
Chromatin profiling in SMCHD1-deficient cells. (**A**) Venn diagrams for comparison of peaks enriched in controls, BAMS and FSHD2 hiPSC-derived MFs for CTCF or H3K27me3. (**B**) GO terms for BPs corresponding to peaks enriched in CTCF or H3K27me3 in BAMS cells (cyan) or FSHD2 (red) MFs. Bars correspond to the number of genes corresponding to the different BPs. Light gray bars on the right represent the enrichment score (log_10_FDR) for each GO term. (**C**) Venn diagrams for comparison of peaks enriched in controls, BAMS and FSHD2 hiPSC-derived NCSCs for CTCF or H3K27me3. (**D**) GO terms for BPs corresponding to peaks enriched in CTCF or H3K27me3 in NCSCs in BAMS (cyan) or FSHD2 (red) cells. Bars correspond to the number of genes corresponding to the different BPs. Light gray bars on the right represent the enrichment score (log_10_FDR) for each GO term.

Given the different roles attributable to SMCHD1 and the identification of changes in the expression of genes associated with BPs relevant to BAMS or FSHD2 respective phenotype in hiPSC-derived NCSCs and MFs (42,56), we then analyzed the top 15 BPs associated with peaks found in patient’s cells for the different chromatin marks (Figure 6). We observed similar BPs for H3K4me3 in BAMS and FSHD2 MFs or NCSCs and H3K27me3 peaks in NCSCs. Most BPs are associated with RNA processing and telomere or chromatin regulation (Supplementary Figure S15A–D). Compared to NCSCs, the top 15 BPs are different in BAMS and FSHD2 MFs with many BPs related to development and differentiation (Supplementary Figure S15A and B). In BAMS MFs or NCSCs, CTCF peaks mostly correspond to BPs related to cell migration and motility or tissue differentiation (Figure 6A–D). In FSHD2 MFs and NCSCs, CTCF peaks are largely associated with BPs related to neuromuscular processes (striated muscle contraction or structure, actin filament processes) (Figure 6A–D). H3K27me3 peaks in BAMS and FSHD2 MFs and NCSCs correspond to loci encoding genes involved in developmental processes, tissue morphogenesis, pattern specification and cell migration (Figure 6A–D).

Noticeably, in FSHD2 MFs, the top 15 H3K27me3 and CTCF peaks are found at loci encoding genes involved in skeletal muscle or neuromuscular function, muscle contraction and actin filament-based processes, all consistent with the disease’s clinical manifestations.

### SMCHD1 regulates tissue-specific differentiation pathways

**Figure 7.**
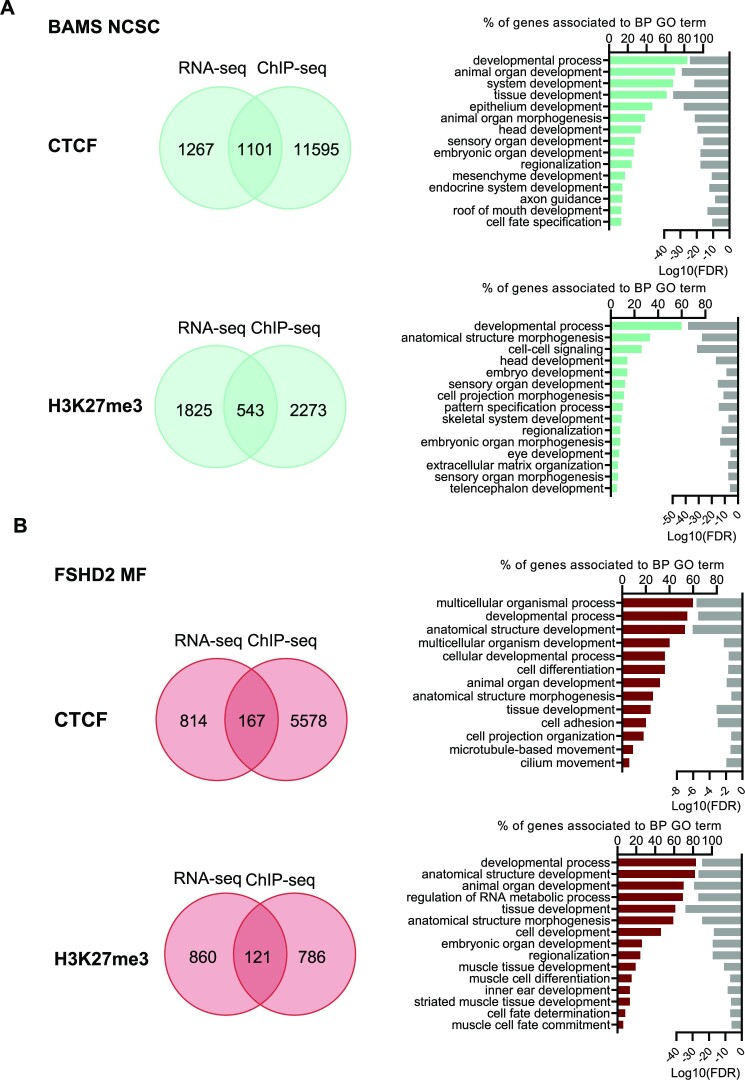
Comparison between chromatin peaks and DEGs in NCSCs and MFs from BAMS and FSHD2 patients. (**A**) Venn diagrams (left) and GO terms for BPs (right) of peaks specifically enriched in CTCF or H3K27me3 in BAMS NCSCs (cyan). (**B**) Venn diagrams (left) and GO terms for BPs (right) of peaks specifically enriched in CTCF or H3K27me3 in FSHD2 MFs (red). For GO terms, bars correspond to the number of genes corresponding to the different BPs. Light gray bars on the right represent the enrichment score (log_10_FDR) for each GO term.

Giving the striking overlap between BPs associated with DEGs (42,56) and chromatin profiling in hiPSC-derived cells, we analyzed more carefully the overlap between CTCF or H3K27me3 peaks and DEGs. We specifically focused on MFs in FSHD2 cells and NCSCs in BAMS cells (Figure 7 and Supplementary File S5).

In BAMS NCSCs, BPs corresponding to the overlap between DEGs and CTCF- or H3K27me3-enriched peaks are consistent with those identified by RNA-seq in this tissue (42). They include developmental processes and embryonic development, head, eye and sensory organ development, or extracellular matrix organization that are relevant to the clinical manifestations that characterize this developmental syndrome (Figure 7A). Among H3K27me3 peaks that are specific to BAMS NCSCs, we noticed peaks corresponding to *HOX* genes (*HOXB1*, *HOXB2*, *HOXC4*, *HOXA3*), all associated with pattern specialization processes and development.

In FSHD2 MFs, BPs correspond to genes associated with developmental and differentiation processes. Among them, several BPs include transcription factors involved in differentiation pathways. In FSHD2 MFs, several DEGs associated with changes in H3K27me3 peaks are also associated with skeletal muscle tissue development, muscle cell fate commitment and striated muscle differentiation (Figure 7B). Furthermore, by considering GO, KEGG or Reactome, we noticed a significant overrepresentation of genes related to calcium (calcium signaling, calcium ion binding, calcium ion transmembrane transport) in cells from FSHD2 patients carrying a mutation in *SMCHD1*, compared to controls.

## DISCUSSION

Originally identified as a regulator of variegation in the mouse (1), Smchd1 is required for proper X inactivation and regulates large gene clusters such as *Hox* or *Pcdh* loci and imprinted gene chromatin in this species (3,4,6–8,13–16). At the two-cell stage, Smchd1 shields against active Tet-dependent DNA demethylation (57) and plays a predominant role in autosomal imprinting through a maternal effect (13,58). However, the role of this protein in human tissues remains only partially understood despite its implication in several distinct rare genetic diseases (21,23–25). In mice, Smchd1 KO is fully penetrant. In BAMS patients, the majority of *SMCHD1* mutations cause a gain of function of the ATPase activity (29). Mutations in FSHD2 act on protein dosage or cause a loss of function (29). Importantly, key differences between mouse and human are the absence of BAMS and FSHD2-like phenotype in mouse models and the lack of a clear evidence for X inactivation defect in BAMS and FSHD2 patients (30), as both sexes are equally affected.

To define SMCHD1 role in the epigenetic regulation of the human genome and its implication in FSHD and BAMS, we performed genome-wide DNAme and chromatin profiling in cells from patients carrying a heterozygous mutation in this gene. By focusing on the role of this protein in the regulation of autosomes, we show here that SMCHD1 shapes chromatin at different levels depending on functional genomic features. As in the mouse, SMCHD1 contributes to *de novo* DNAme but is dispensable for DNAme maintenance in human cells. Its occupancy is not correlated with the level of DNAme (8,30,57). In human cells, SMCHD1 is involved in the distribution of repressive marks (DNAme, K3K27me3) at heterochromatin. We further show that SMCHD1 might also shield against the deposition of H3K27me3 suggesting a role in chromatin insulation. Importantly, SMCHD1 also regulates DNAme and H3K27me3 deposition at euchromatin loci, orchestrating the expression of genes involved in development and differentiation circuits.

Our results further suggest that SMCHD1 might impact gene expression in two ways: (i) by acting on CTCF and H3K27me3 enrichment, changing the chromatin context at a number of loci, or (ii) by regulating loci encoding master transcription factors required for cell fate determination and differentiation. In addition, SMCHD1 contributes to chromatin insulation by protecting against repressive chromatin and contributes to D4Z4 boundary activity (45,59). We further uncovered here that in human cells, multiple roles are attributable to SMCHD1, either as a *cis-*activator (D4Z4–DR1, *SEMA5A*) or as a moderate (*HOXB2*, *NCAM2*, *WASH7PL*) to strong (*HOXA13*, *HOXB6*, *HOXC4/C5/C6*, *BET1L*) repressor of gene expression. As Smchd1 protects against Tet-dependent demethylation (57), it remains to be determined whether the increased proportion of hypomethylated CpGs in BAMS and FSHD2 cells is due to SMCHD1 inability to counteract active demethylation or to target *de novo* methylation during cell fate transition and cell lineage specification.

Autosomal dominant *SMCHD1* mutations impact stem cell properties as well as postnatal health (60). The diverse phenotypes of *SMCHD1*-mutated patients and animal models might be the consequence of pleiotropic effects of the protein. These pleiotropic effects might be triggered by dosage-dependent or functional chromatin state changes at regulatory regions with cell-to-cell differences. In human cells, these stochastic changes might contribute to the variegated expression of gene required for development and differentiation in space and time. This variegation in expression might, in particular, concern master transcription factors required for cell differentiation as evidenced by comparing H3K27me3 and CTCF profiles. Changes in the epigenetic signature of several predicted SMCHD1-dependent targets, including gene promoters and enhancers, might induce promiscuous or precocious activation/repression of genes controlled or located at distance from these regulatory elements.

In patients carrying a heterozygous *SMCHD1* mutation, SMCHD1 deficiency is less drastic than in KO mice that die *in utero*. However, *SMCHD1* mutations impact NCSCs and skeletal muscle differentiation. Based on our results from patient’s cells, we hypothesize that titration of functional SMCHD1 homodimers alters DNA or H3K27 methylation and CTCF-dependent chromatin organization at various loci. Several of these loci are associated with striated muscle and NCSC differentiation or function. These changes might in turn modify the expression of genes implicated in development, cell fate transition and tissue differentiation in rare genetic diseases linked to heterozygous *SMCHD1* mutations. The specific molecular mechanisms by which DNAme or H3K27me3 regulates the selection of CTCF sites involved in cell differentiation, among the thousands present, are still not fully understood (61,62). We now need to understand how SMCHD1 regulates DNAme or H3K27me3 deposition and how SMCHD1 and CTCF might cooperate in guiding the organization and topology of the human genome.

Our study, along with previous research in animal models (6,55), confirms the critical role of SMCHD1 in shaping the mammalian genome and sheds light on the consequences of *SMCHD1* mutations in rare genetic diseases. Our findings demonstrate the link between SMCHD1 function and cell differentiation and development, providing new avenues for investigating diseases associated with mutations in this gene. Our results emphasize the importance of characterizing SMCHD1’s functional domains to better understand the effects of germline mutations on genetic diagnostics and therapeutic interventions. Additionally, our gene expression and chromatin profiling data consistently demonstrate that hiPSC-derived innervated fibers from patient cells provide an accurate model for studying functional muscle defects in cells from FSHD patients.

## Supplementary Material

gkad523_Supplemental_Files

## Data Availability

Raw data were deposited at the NCBI Gene Expression Omnibus (https://www.ncbi.nlm.nih.gov/geo/) with the following accession numbers. Raw RNA-seq data and raw count matrix: GSE174604. DNAme data: GSE175527. Raw ChIP-seq data in MFs and raw count matrix record: GSE174769. Raw ChIP-seq data in NCSCs and raw count matrix record: GSE179986.
