# Supplementary material for: In skeletal muscle and neural crest cells, SMCHD1 regulates biological pathways relevant for Bosma syndrome and facioscapulohumeral dystrophy phenotype
Source: Nucleic Acids Res. 2023 Jun 19;51(14):7269–87. doi: 10.1093/nar/gkad523 (PMC10415154; doi:10.1093/nar/gkad523)
Supplement: gkad523_Supplemental_Files [file gkad523_supplemental_files.zip › Laberthonniere et al. Sup info.pdf]

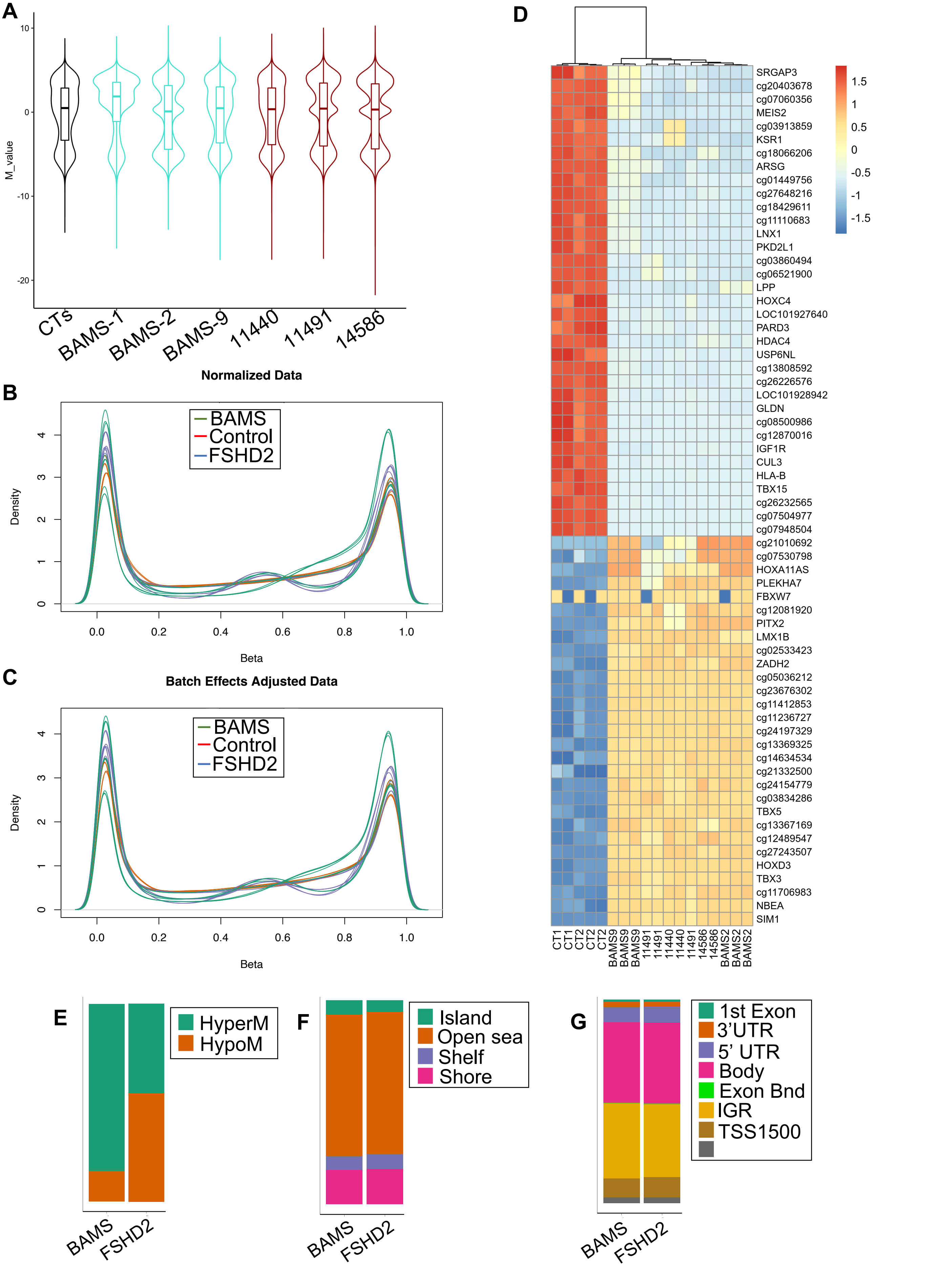

Laberthonnière et al.

Figure S1

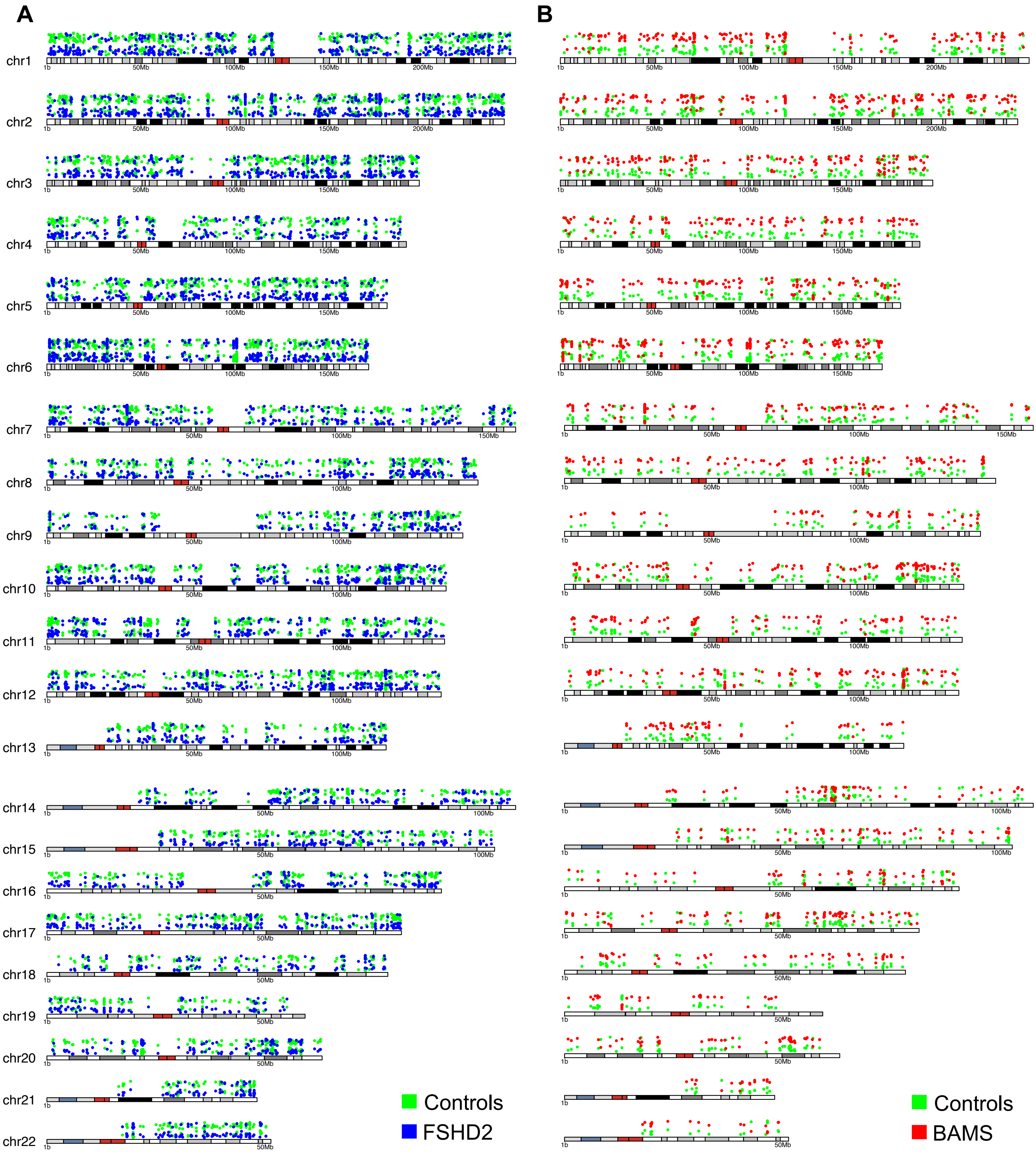

Laberthonnière et al.

Figure S2

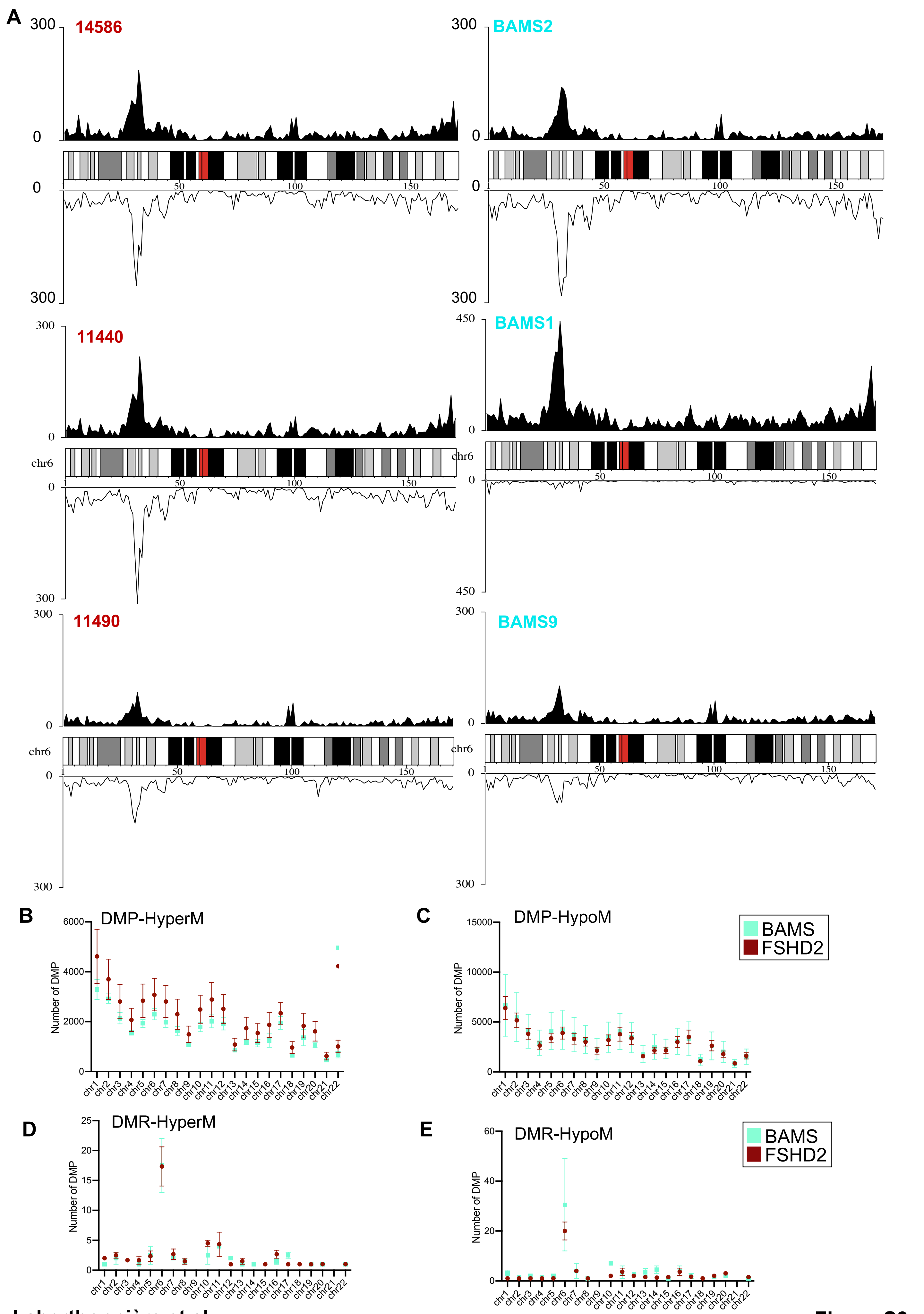

Laberthonnière et al.

Figure S3

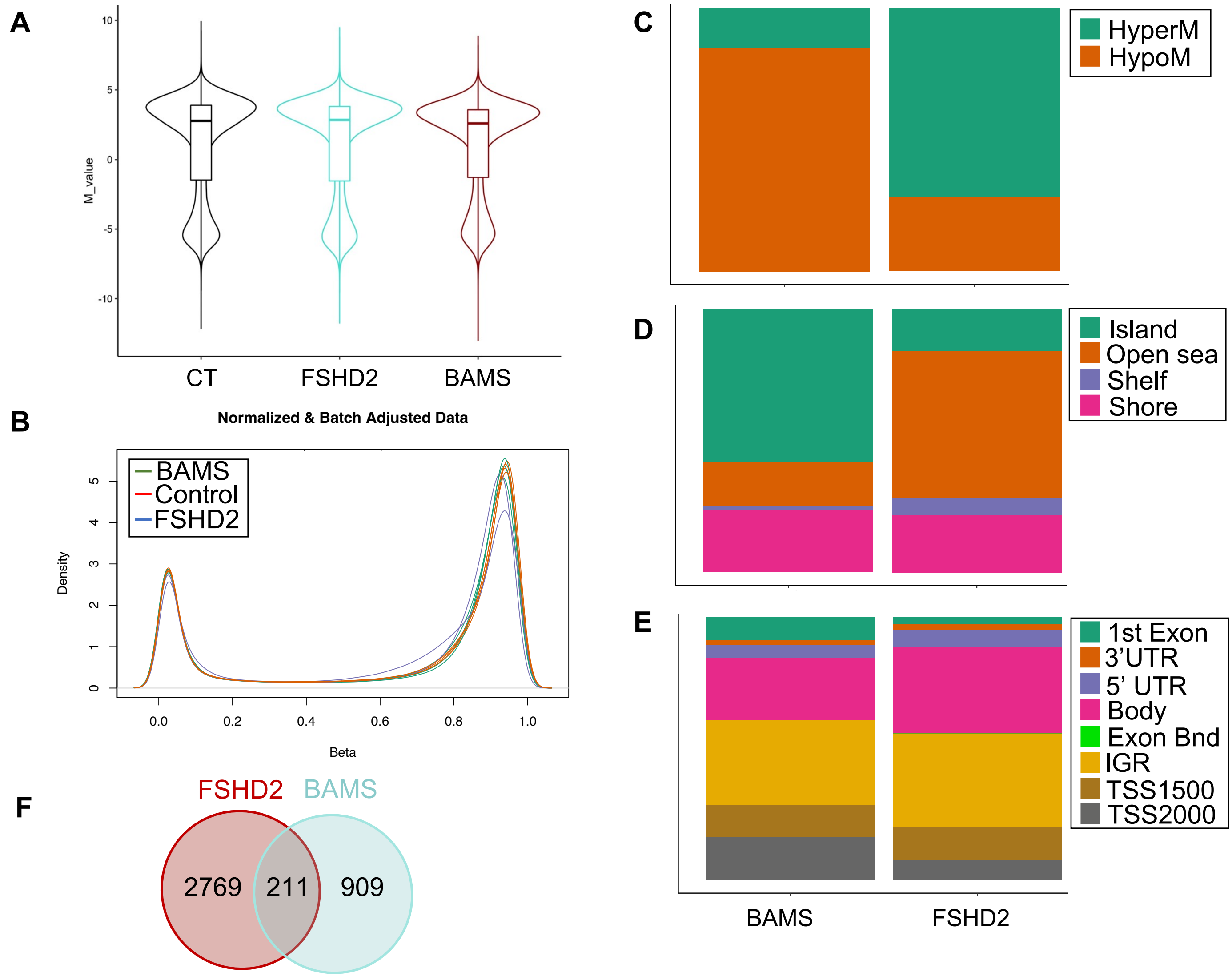

Laberthonnière et al.

Figure S4

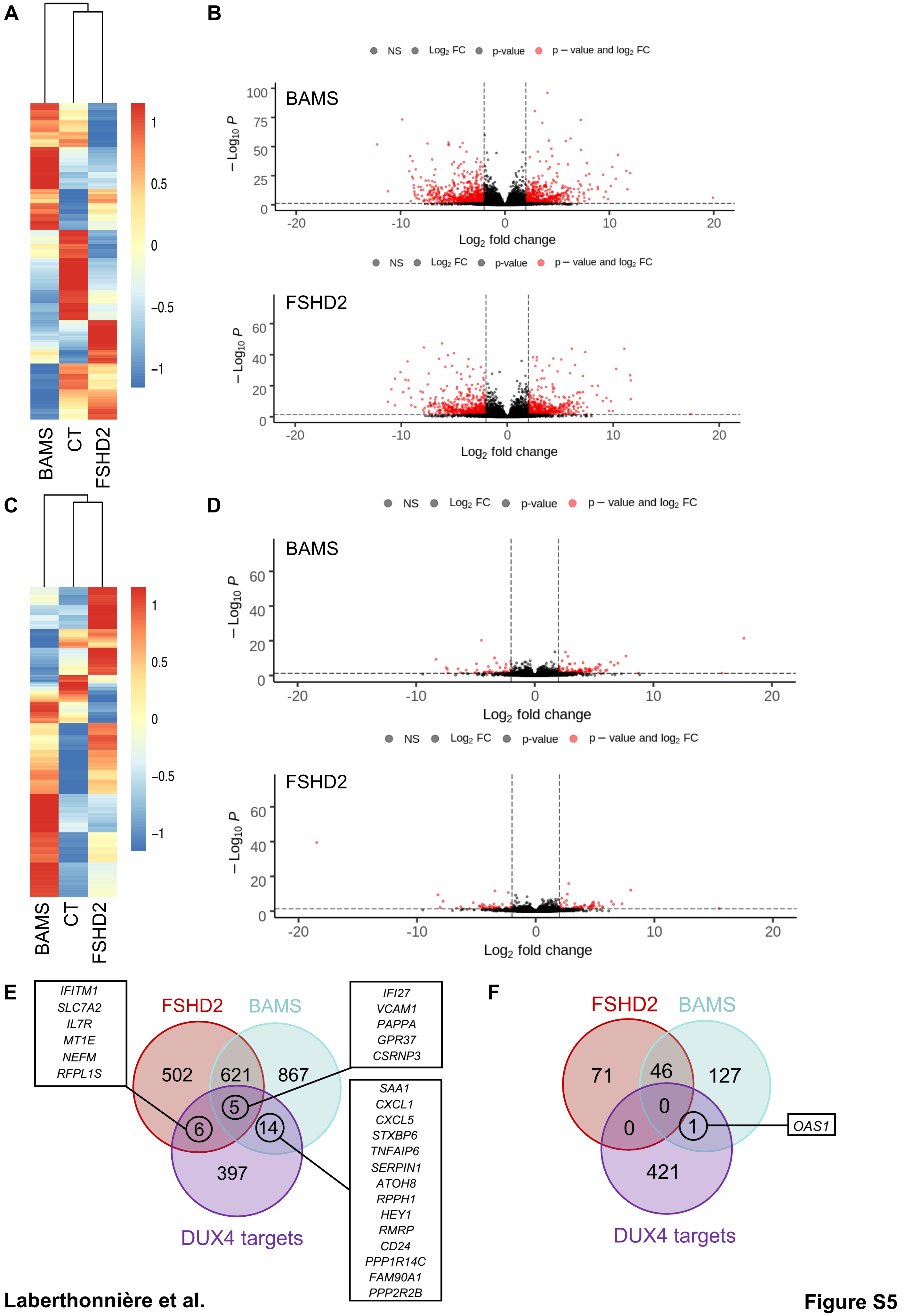

Laberthonnière et al.

Figure S5

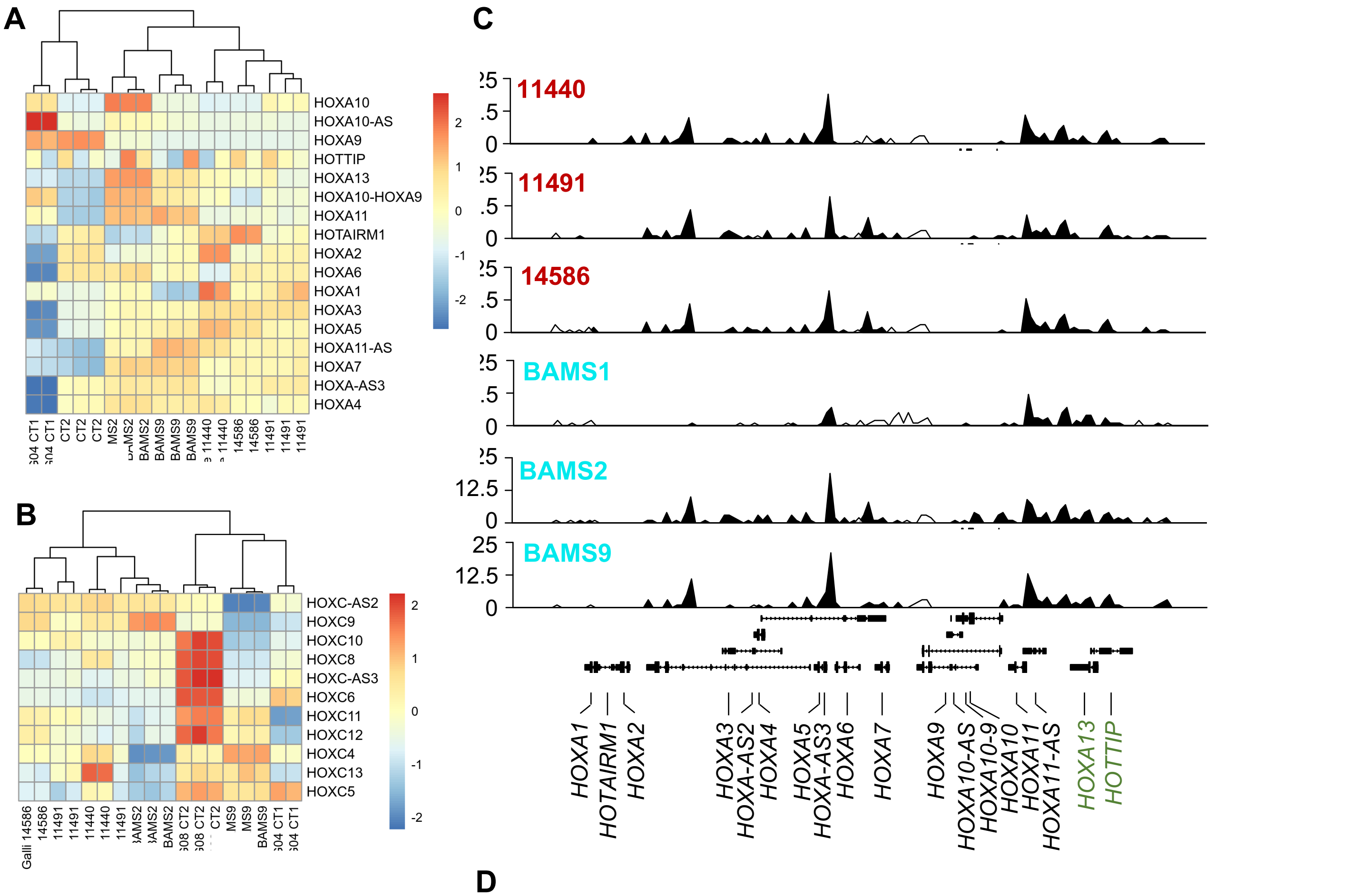

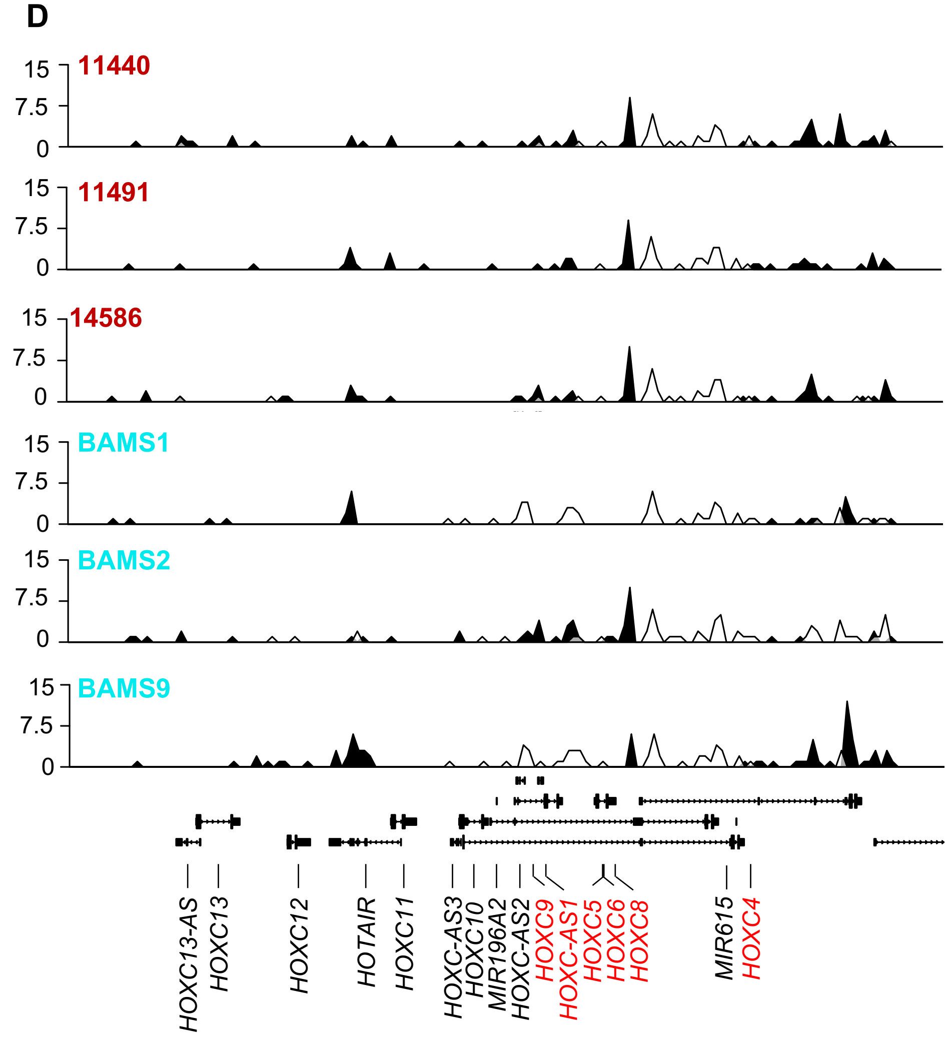

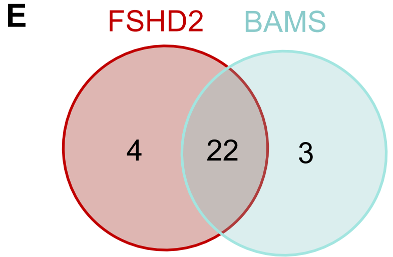

F

	FSHD2	BAMS
HOTTIP	-10.07	-8.81
HOXA13	-4.08	-6.47
HOXB-AS2	6.89	7.19
HOXB-AS3	10.28	10.28
HOXB3	5.87	6.04
HOXB4	9.79	9.65
HOXB5	11.05	10.81
HOXB6	11.66	11.73
HOXB7	11.59	11.62
HOXB8	9.36	10.14
HOXB9	6.17	9.47
HOXC-AS1	2.59	3.39
HOXC4	2.85	2.98
HOXC5	3.71	3.58
HOXC6	3.06	2.78
HOXC8	6.41	6.03
HOXC9	2.80	4.07
HOXD1	7.19	8.32
HOXD3	3.95	4.52
HOXD10	-5.55	-6.12
HOXD11	-5.25	-6.14
HOXD13	-3.79	-7.95

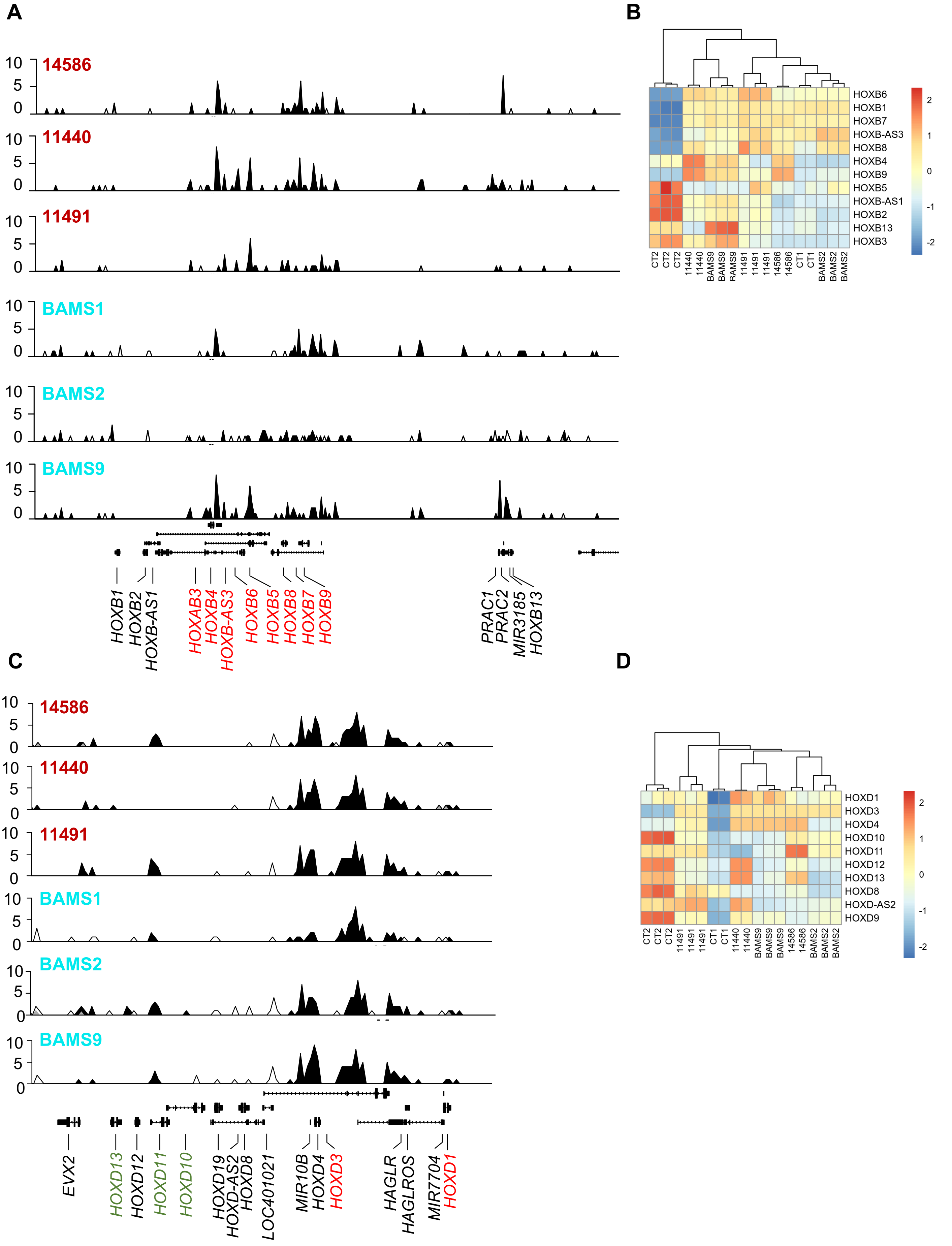

Laberthonnière et al.

Figure S7

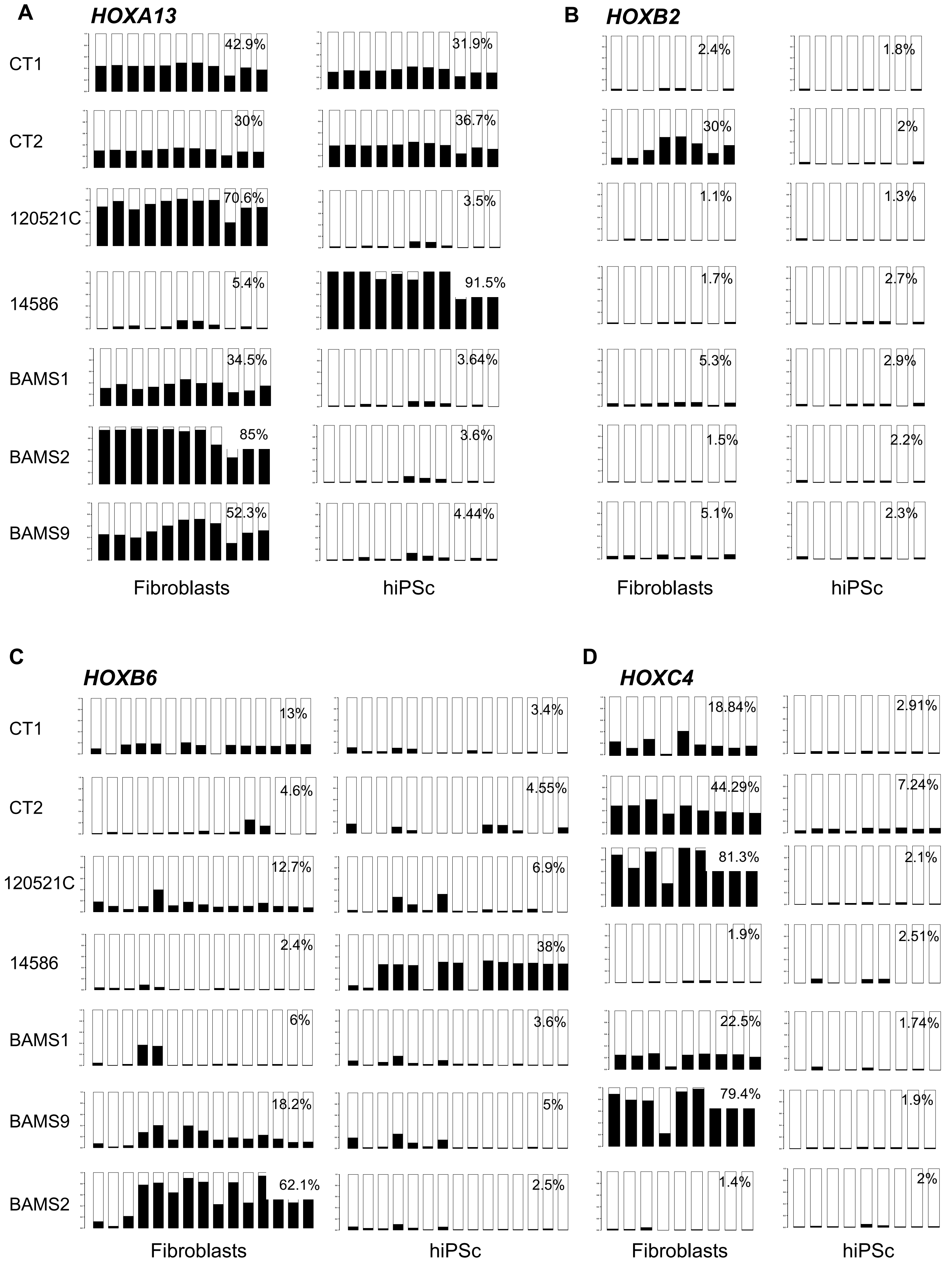
A
HOXA13
CT1
42.9%
31.9%
CT2
30%
36.7%
120521C
70.6%
3.5%
14586
5.4%
91.5%
BAMS1
34.5%
3.64%
BAMS2
85%
3.6%
BAMS9
52.3%
4.44%
Fibroblasts
hiPSc
B
HOXB2
2.4%
1.8%
30%
2%
1.1%
1.3%
1.7%
2.7%
5.3%
2.9%
1.5%
2.2%
5.1%
2.3%
Fibroblasts
hiPSc
C
HOXB6
CT1
13%
3.4%
CT2
4.6%
4.55%
120521C
12.7%
6.9%
14586
2.4%
38%
BAMS1
6%
3.6%
BAMS9
18.2%
5%
BAMS2
62.1%
2.5%
Fibroblasts
hiPSc
D
HOXC4
18.84%
2.91%
44.29%
7.24%
81.3%
2.1%
1.9%
2.51%
22.5%
1.74%
79.4%
1.9%
1.4%
2%
Fibroblasts
hiPSc

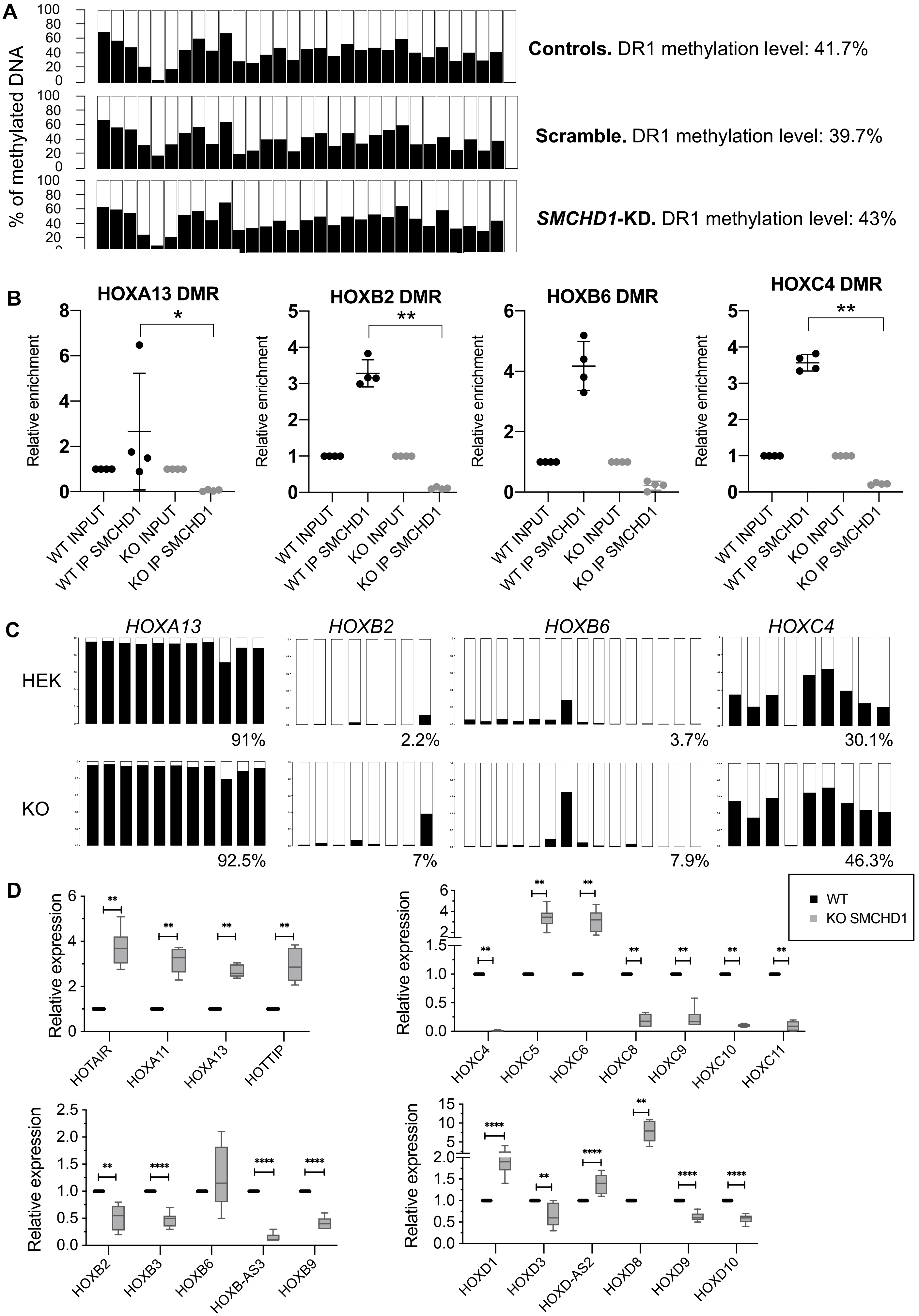

Laberthonnière et al.

Figure S9

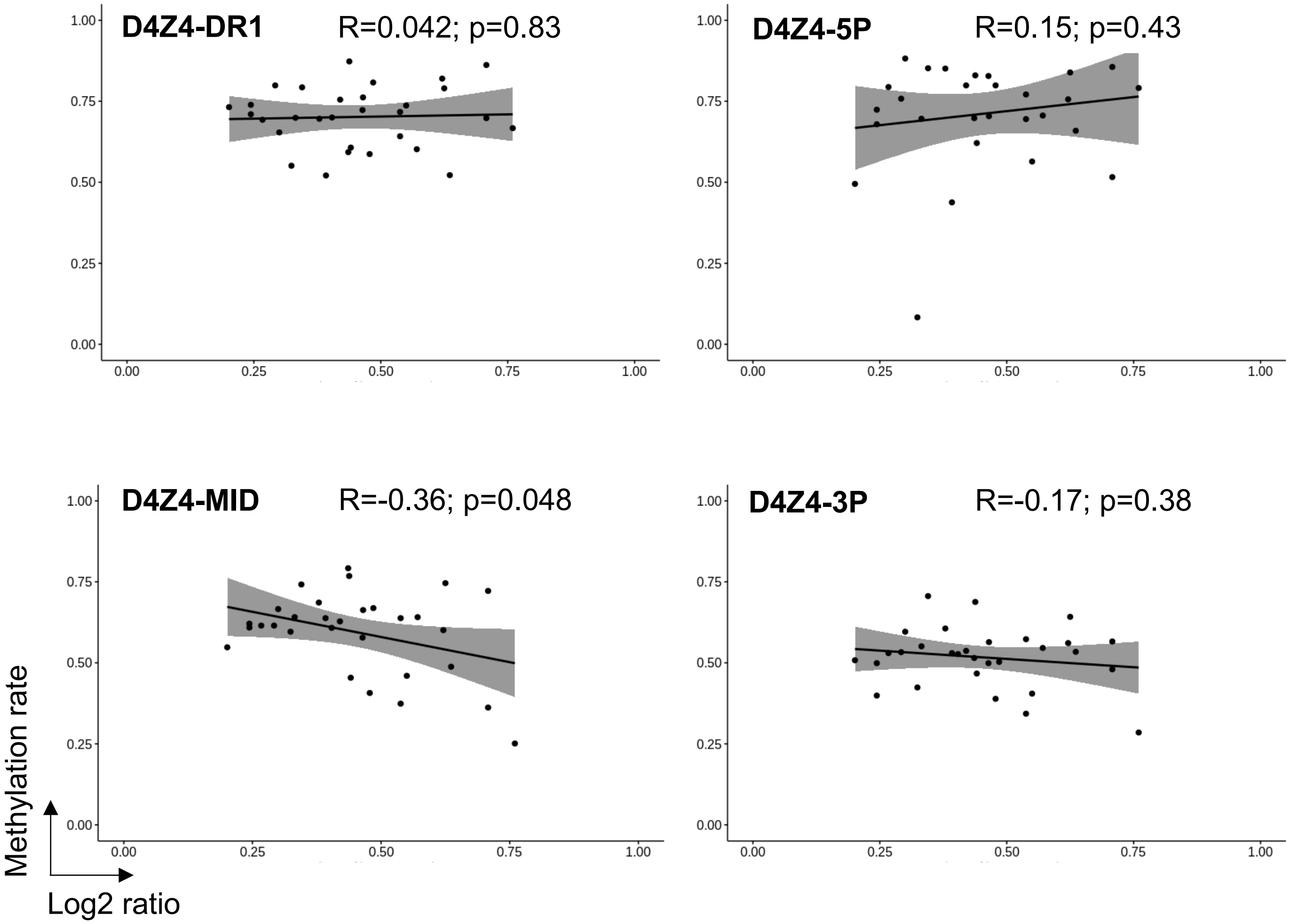

Laberthonnière et al.

Figure S10

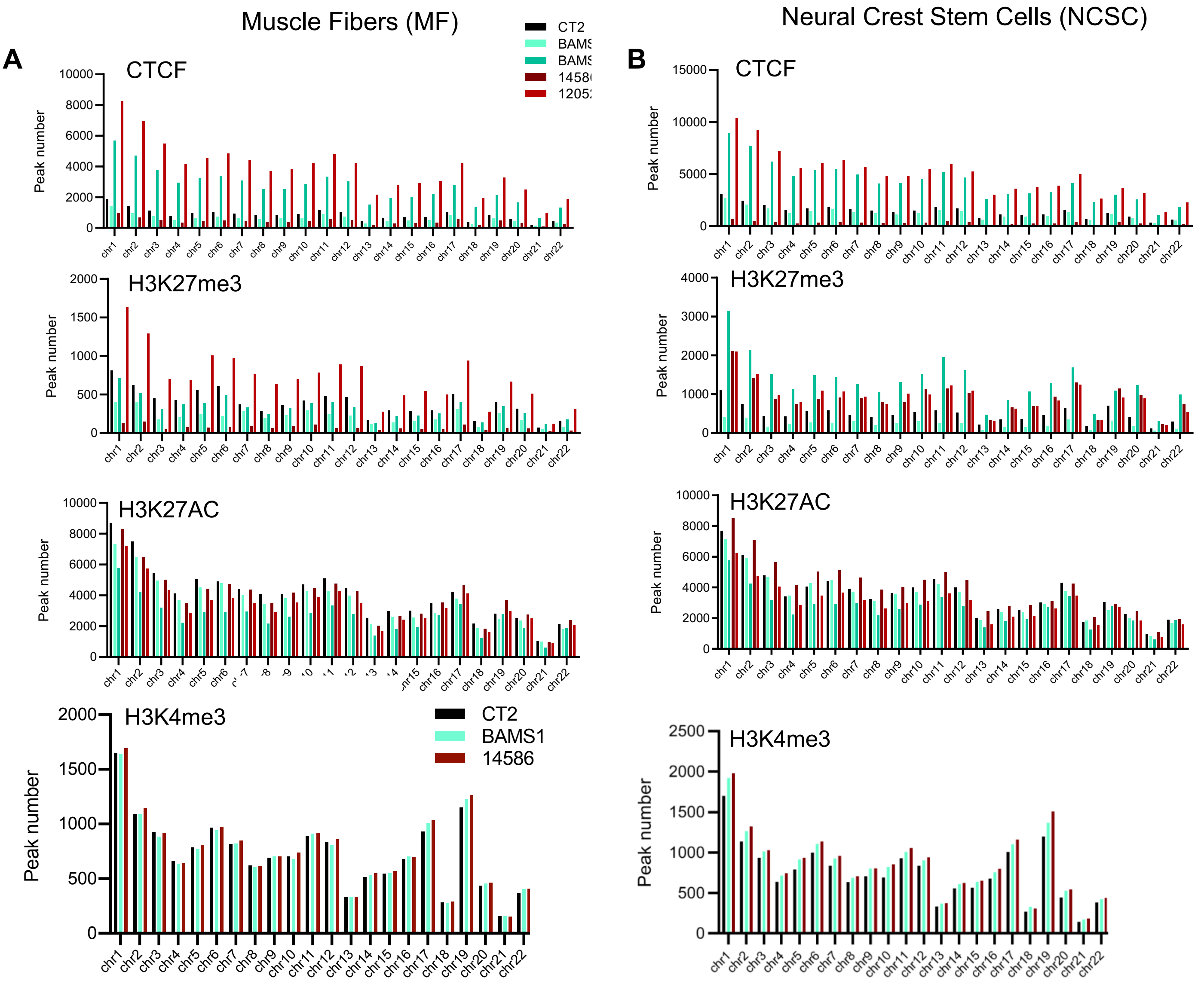
Muscle Fibers (MF)
Neural Crest Stem Cells (NCSC)
A
B
CTCF
H3K27me3
H3K27AC
H3K4me3
Peak number
CT2
BAMS1
14586

Figure S11

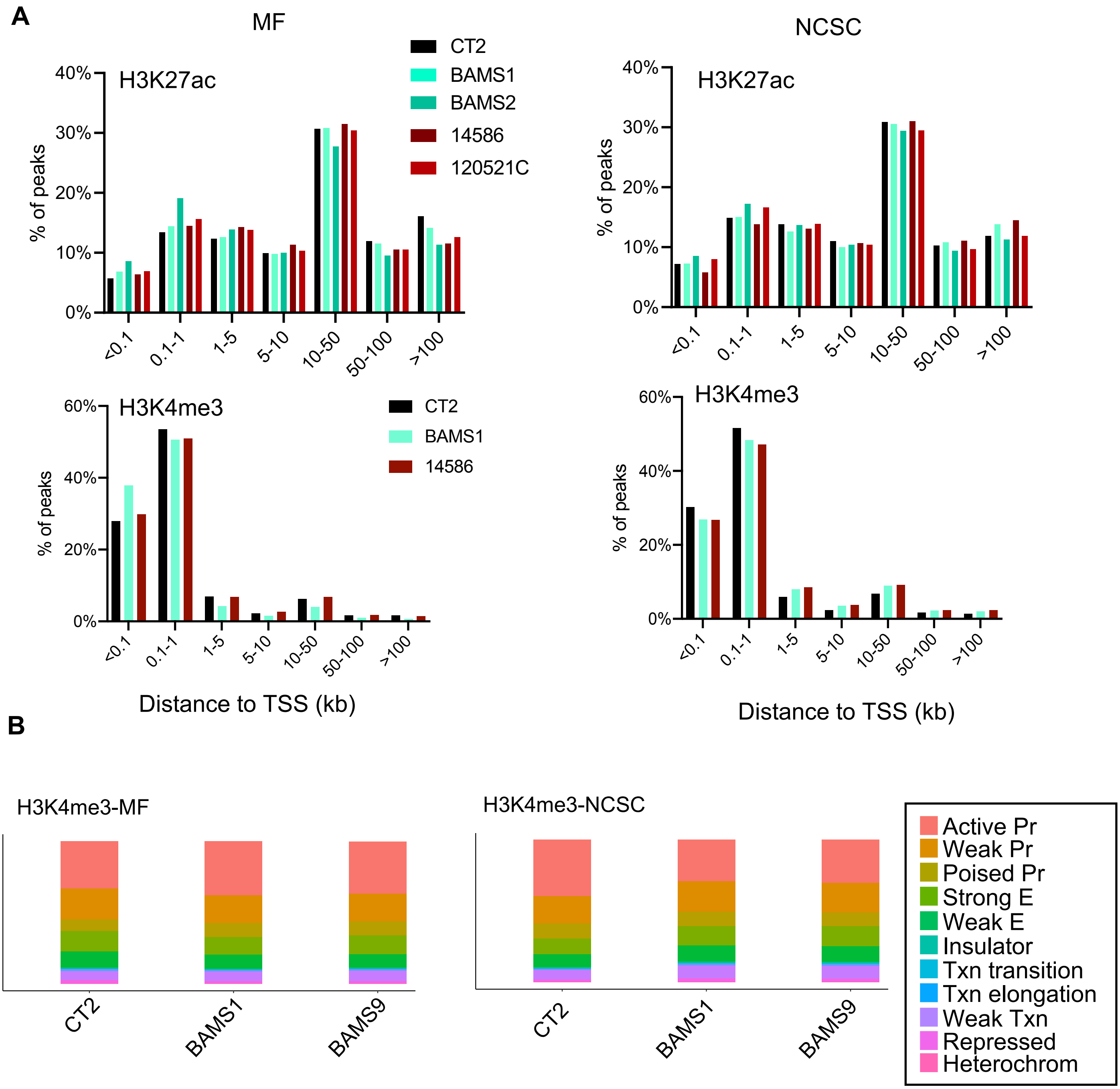

Laberthonnière et al.

Figure S12

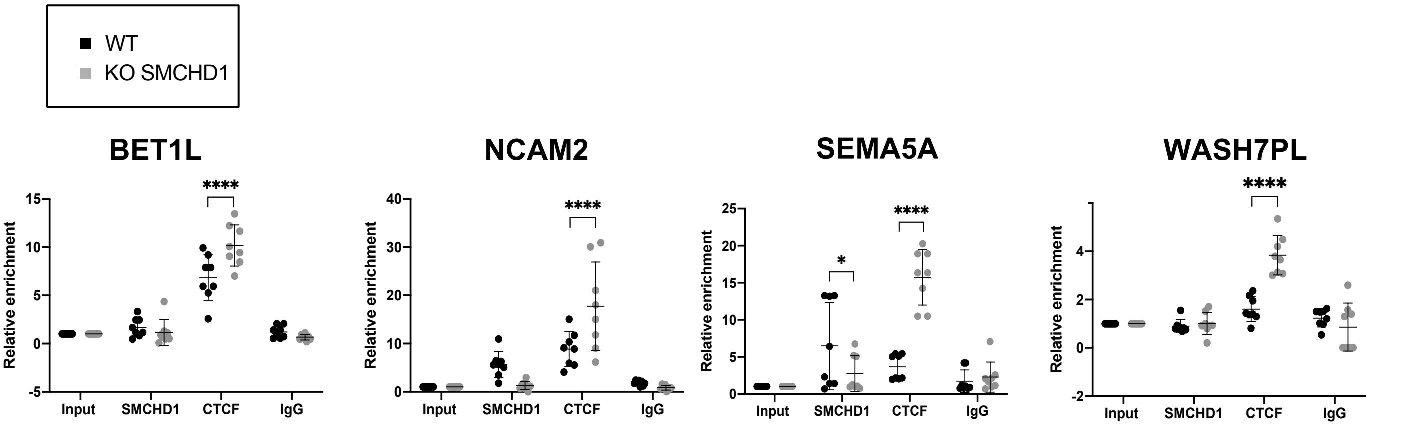

Laberthonnière et al.

Figure S13

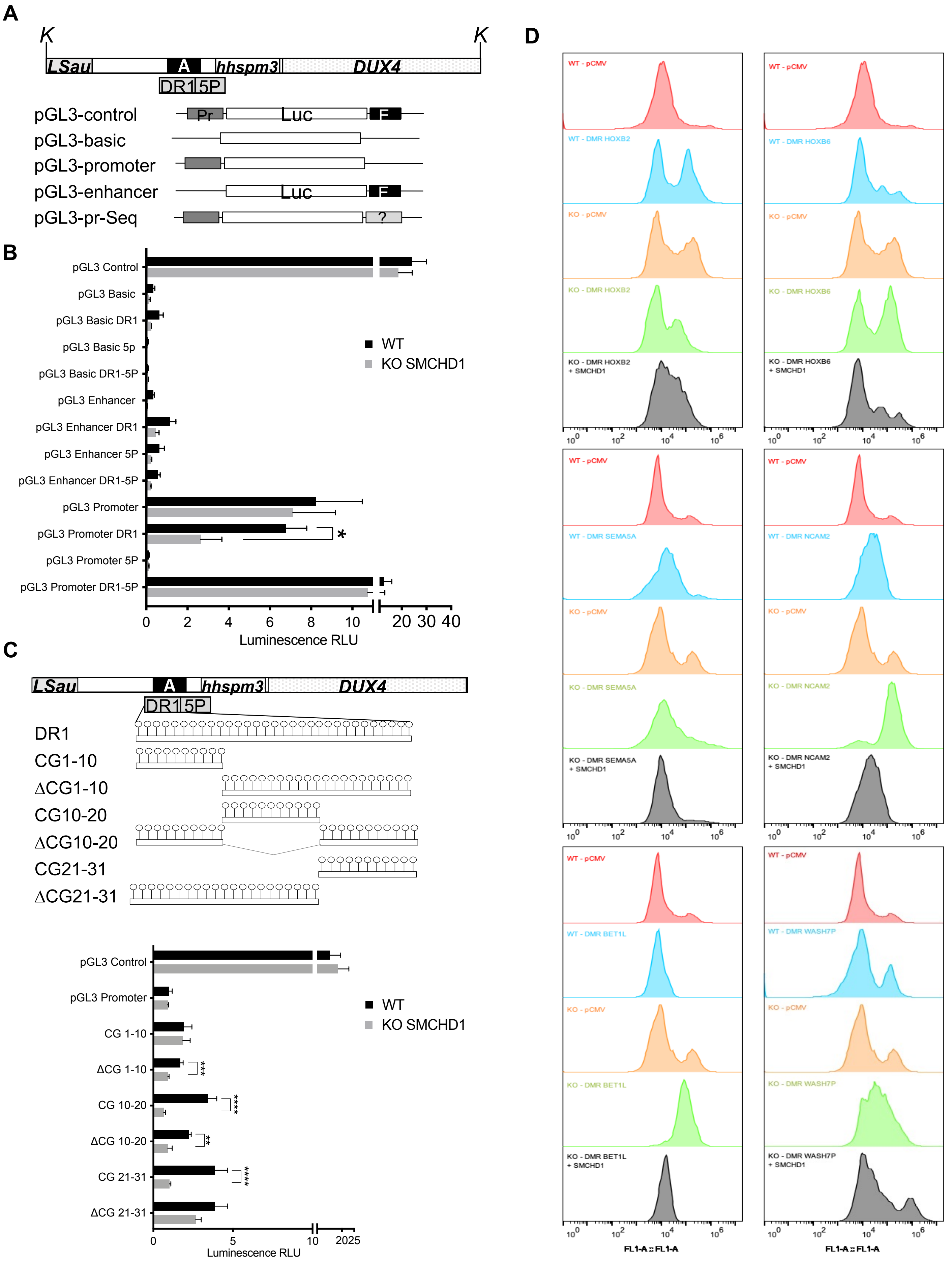

A
K
K
LSau
A
hhspm3
DUX4
DR1 5P
pGL3-control
Pr
Luc
E
pGL3-basic
pGL3-promoter
pGL3-enhancer
pGL3-pr-Seq
?
B
pGL3 Control
pGL3 Basic
pGL3 Basic DR1
pGL3 Basic 5p
pGL3 Basic DR1-5P
pGL3 Enhancer
pGL3 Enhancer DR1
pGL3 Enhancer 5P
pGL3 Enhancer DR1-5P
pGL3 Promoter
pGL3 Promoter DR1
pGL3 Promoter 5P
pGL3 Promoter DR1-5P
WT
KO SMCHD1
Luminescence RLU
C
DR1
CG1-10
ΔCG1-10
CG10-20
ΔCG10-20
CG21-31
ΔCG21-31
pGL3 Control
pGL3 Promoter
CG 1-10
ΔCG 1-10
CG 10-20
ΔCG 10-20
CG 21-31
ΔCG 21-31
WT
KO SMCHD1
Luminescence RLU
D
WT - pCMV
WT - DMR HOXB2
KO - pCMV
KO - DMR HOXB2
KO - DMR HOXB2 + SMCHD1
WT - DMR HOXB6
KO - DMR HOXB6
KO - DMR HOXB6 + SMCHD1
WT - DMR SEMA5A
KO - DMR SEMA5A
KO - DMR SEMA5A + SMCHD1
WT - DMR NCAM2
KO - DMR NCAM2
KO - DMR NCAM2 + SMCHD1
WT - DMR BET1L
KO - DMR BET1L
KO - DMR BET1L + SMCHD1
WT - DMR WASH7P
KO - DMR WASH7P
KO - DMR WASH7P + SMCHD1
FL1-A :: FL1-A

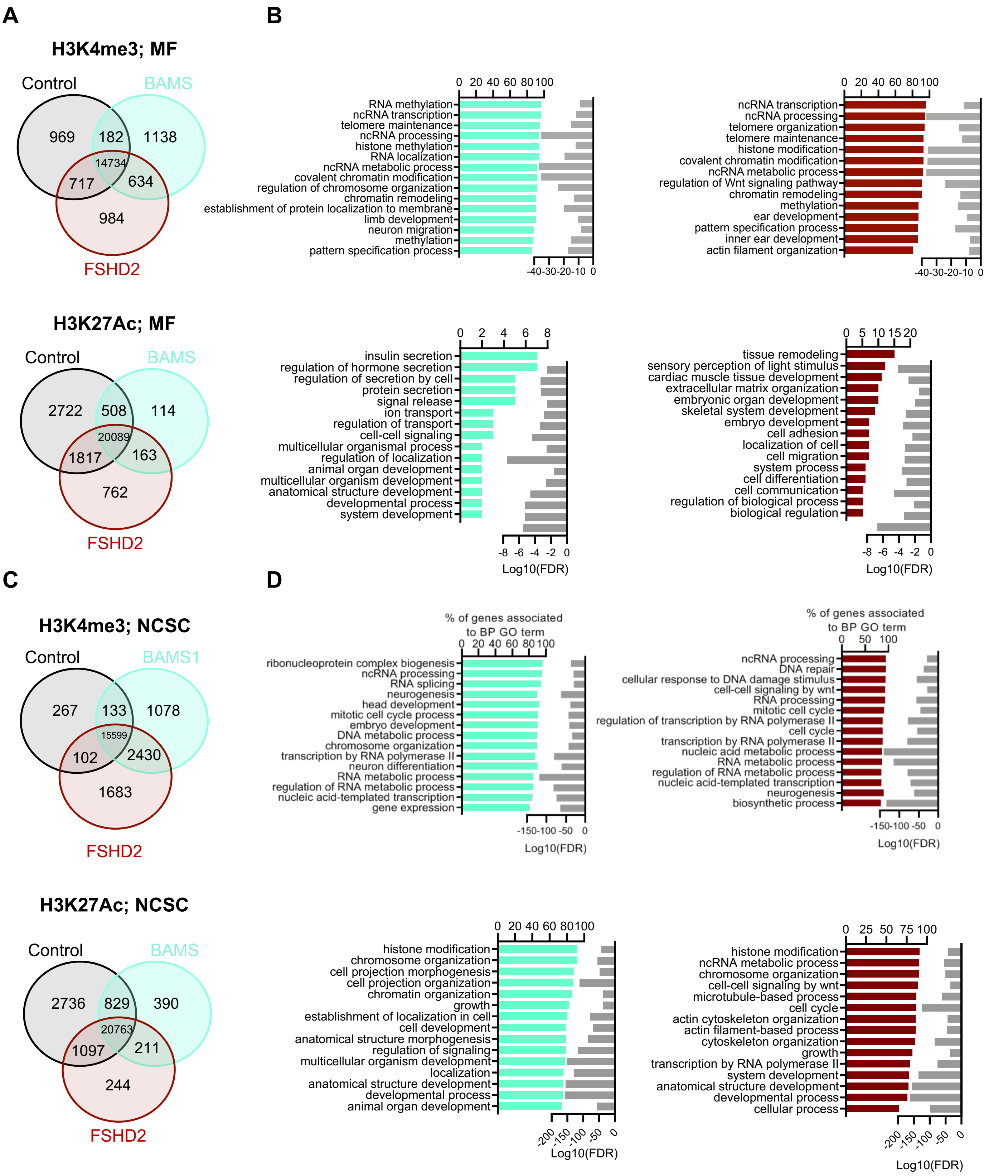
A
B
H3K4me3; MF
Control
BAMS
FSHD2
969
182
1138
14734
717
634
984
RNA methylation
ncRNA transcription
telomere maintenance
ncRNA processing
histone methylation
RNA localization
ncRNA metabolic process
covalent chromatin modification
regulation of chromosome organization
chromatin remodeling
establishment of protein localization to membrane
limb development
neuron migration
methylation
pattern specification process
ncRNA transcription
ncRNA processing
telomere organization
telomere maintenance
histone modification
covalent chromatin modification
ncRNA metabolic process
regulation of Wnt signaling pathway
chromatin remodeling
methylation
ear development
pattern specification process
inner ear development
actin filament organization
H3K27Ac; MF
Control
BAMS
FSHD2
2722
508
114
20089
1817
163
762
insulin secretion
regulation of hormone secretion
regulation of secretion by cell
protein secretion
signal release
ion transport
regulation of transport
cell-cell signaling
multicellular organismal process
regulation of localization
animal organ development
multicellular organism development
anatomical structure development
developmental process
system development
Log10(FDR)
tissue remodeling
sensory perception of light stimulus
cardiac muscle tissue development
extracellular matrix organization
embryonic organ development
skeletal system development
embryo development
cell adhesion
localization of cell
cell migration
system process
cell differentiation
cell communication
regulation of biological process
biological regulation
Log10(FDR)
C
D
H3K4me3; NCSC
Control
BAMS1
FSHD2
267
133
1078
15599
102
2430
1683
% of genes associated to BP GO term
ribonucleoprotein complex biogenesis
ncRNA processing
RNA splicing
neurogenesis
head development
mitotic cell cycle process
embryo development
DNA metabolic process
chromatin organization
transcription by RNA polymerase II
neuron differentiation
RNA metabolic process
regulation of RNA metabolic process
nucleic acid-templated transcription
gene expression
Log10(FDR)
% of genes associated to BP GO term
ncRNA processing
DNA repair
cellular response to DNA damage stimulus
cell-cell signaling by wnt
RNA processing
mitotic cell cycle
regulation of transcription by RNA polymerase II
cell cycle
transcription by RNA polymerase II
nucleic acid metabolic process
RNA metabolic process
regulation of RNA metabolic process
nucleic acid-templated transcription
neurogenesis
biosynthetic process
Log10(FDR)
H3K27Ac; NCSC
Control
BAMS
FSHD2
2736
829
390
20763
1097
211
244
histone modification
chromosome organization
cell projection morphogenesis
cell projection organization
chromatin organization
growth
establishment of localization in cell
cell development
anatomical structure morphogenesis
regulation of signaling
multicellular organism development
localization
anatomical structure development
developmental process
animal organ development
Log10(FDR)
histone modification
ncRNA metabolic process
chromosome organization
cell-cell signaling by wnt
microtubule-based process
cell cycle
actin cytoskeleton organization
actin filament-based process
cytoskeleton organization
growth
transcription by RNA polymerase II
system development
anatomical structure development
developmental process
cellular process
Log10(FDR)

Supplementary information

Table S1: List of primary fibroblasts and hiPSCS clones.

BAMS-Case 1; BAMS-Case 2 and BAMS-Case 9 were described in (1) All cells are described in (2,3).

AG08498	Healthy	No mutation	1	Male	✓	✓
AG04148	Healthy	No mutation	56	Female	✓	✓
11440	FSHD2	c.2338+4A>G; p.S754*	37	Male	✓	✓
11491	FSHD2	c.5476+3 A>G; r.5477_5547del; p.V1826Gfs*19	66	Female	✓	✓
14586	FSHD2	c.573A>C ; p.Q193P	67	Male	✓	✓
34140	FSHD2	p.L1031	10	Female	✓	✓
BAMS-1	BAMS	c.407A>G p.E136G	5	Male	✓	✓
BAMS-2	BAMS	c.403A>T p.S135C	28	Female	✓	✓
BAMS-9	BAMS	c.1259A>T p.D420V	3	Male	✓	✓

Table S2: Sequence of the primers used for RT-qPCR. Primers for DUX4 and DUX4 targets were described in (4).

HPRT	TGATAGATCCATTCCTATGACTGTAGA	CAAGACATTCTTTCCAGTTAAAGTTG
PPIA	ATGCTGGACCCAACACAAAT	TCTTTCACTTTGCCAAACACC
GAPDH	AGCCACATCGCTCAGACAC	GCCCAATACGACCAAATCC
HOTAIR	ATGAACTGGCGAGAGGTCTG	TTCAACCCCCTCCCCCATAA
HOXA11	GGCGGCTCCAGTGGC	CGCTGAAGAAGAACTCCCGT
HOXA13	GGAACGGCCAAATGTACTGC	GATGGGAGACCACGTCGGG
HOTTIP	TTACGCCCGCAACAAAACAG	CCCTCCTTCCTTCAAACGCT
HOXC4	ATGGCCAGAGGGTTGGAAATTA	CATCCCTGAACACAGTCCGG
HOXC5	CACATGAGCCACGAGACGG	TCCACTTCATCCTGCGGTTC
HOXC6	ATGAATTCGCACAGTGGGGT	TTGATCTGTCGCTCGGTCAG
HOXC8	ACAGTAGCGAAGGACAAGGC	CCAAGGTCTGATACCGGCTG
HOXC9	CCGCAGCTACCCGGACTA	CGTGAATCCAGTTGGCCACG
HOXC10	CCCTCGGAGAGCGAAAAGG	TCAGCCAATTTCCTGTGGTGT
HOXC11	GGCTGAGGAGGAGAACACAAA	GTCGGTCCGTCAGGTTCAG
HOXB2	GAGAGACCGAAATCTCCCCC	AAGGAAGTCAGACACTCGGC
HOXB3	CCTCCCGCAAATCTCCTTGG	CCCTCCTTTGCGCCTCTC
HOXB6	GGTCTGCAAAGGCGCGG	AGGAACTCATTGGGAGGGGA
HOXBAS3	CCCTCCCTCCAAGTCCAGTA	GGATATCGCTGGGTTCCCG
HOXB9	GAGAGGCCGGATCAAACCAA	CTACGGTCCCTGGTGAGGTA
HOXD1	CTCTCTGGAACAACCCCCAC	CTGGAACTCGGAAGCCAACT
HOXD3	GTCGTCATTAATCTGCCACGC	GGCTCCAGGTGACCACAATTA
HOXDAS2	GCGATTCTTACCCGAAGGCT	AGCGCCTAGTGGATTACAGC
HOXD8	CAGTGCTGTGGTGCGAAAAT	ACACTCTGGCCTCGGTTTAC

Table S3: Sequence of the primers used for Sodium bisulfite PCR. Primers for D4Z4 were described and validated in (5,6).

[redacted]				
D4Z4	5P	AAATATGTAGGGAAGGGTGTAAGTT<br>CTTAAATATACCAAACCCTCTCTCC	56°C	341
	MID	ATTTATGAAGGGGTGGAGTTT<br>ATAACCTAAACCAACCRTTCTCTA	56°C	419
	3P	GTTTTGTTGGAGGAGTTTTAGGA<br>CTAAACCTAAAAAACAAAAATCCCA	56°C	237
	DR1	GAAGGTAGGGAGGAAAAG<br>ACTCAACCTAAAAATATACAATCT	56°C	254
HOX	HOXA13	TGTGTTAGTTTATTTTTGGTTATGG<br>CAAACAAACCTACTTATAACTCCTC	56°C	216
	HOXB2	TATTTTAGATTTAATGGTGGTTGGG<br>ACCTCTAAATTTTTCATTCATTAACCTT	56°C	213
	HOXB6	GGGAGATAGTAAATATTTTTTTGT<br>AAAAACCAATAATTCTAACCTCC	58°C	269
	HOXC4	TTTTTATTTGTTTGGTTTAGTTGGG<br>CTTTTCCAAAAATCTCCATTCATAA	56°C	273

Table S4: Position of genomic fragment, related to the Hg38 assembly for reporter gene assays.

BET1L	Chr 11	190011	190271
HOXA13	Chr 7	27201926	27202927
HOX B2	Chr 17	48544650	48545353
HOXB6	Chr 17	48603749	48604273
HOX C4	Chr 12	54053160	54054306
NCAM2	Chr 21	21003843	21003903
SEMA5A	Chr 5	9339379	9339419
WASH7PL	Chr 1	10071	10271

Table S5: Sequence of primers used for ChIP

BET1L	TTGCTTGCAGTGTAGTCGGG	GGGAGGAATCCCAGCAAGAT
CHR5	GGAGTTGGGGAAGCTAGGAA	GATCATCCGTGGCTTGAGAT
D4Z4-3’	CTCAGCGAGGAAGAATACCG	ACCGGGCCTAGACCTAGAAG
D4Z4-5’	ACGACGGAGGCGTGATTT	AGTGTGGCCGGTTTGGAA
D4Z4-DR1	CCCGCCTCCGGGAAAAC	GGGATGTGCGGTCTGTGAA
D4Z4-Mid	TCATGAAGGGGTGGAGCCTG	TCCAAACGAGTCTCCGTCGC
NCAM2	CCAGACACCTCATTAGACAGCA	GTGCCCATCCTAAGCCTCTTG
SEMA5A	ATGTCCTAGAGGGGCAAAGC	AATGCAGACGCAAGAGCCAT
WASH7P	CGCCTTCAGAGTACCACCG	TCAGCACAGACCCGGAGA
DMR HOXA13	GGCGGTGTTTACCGACTCTT	GTCGTCTGTGGAGCTGAGAC
DMR HOXC4/5/6	CCCATCCAACATCCACTCCAG	CTCCATTCATGGCGATCGTG
DMR HOXB2	AAACACCAGAGGACCACGAC	GCATTGGGGAGAGAGCATGT
DMR HOXB6	CAGGCCCTATAGAAACCAGGAC	TTCTCTTGCCTGGTGCGGAT

Table S6. Distribution of DMPs in samples groups and individual fibroblasts samples.

[redacted]								
**HypoM**	5057<br>17.13%	9415<br>5.4%	100538<br>72.05%	37215<br>54.41%	29616<br>55.14%	78631<br>56.55%	43793<br>63.05%	70703<br>54.72%
**HyperM**	26514<br>82.87%	165059<br>94.60%	38997<br>27.95%	31179<br>45.59%	24094<br>44.86%	60412<br>43.45%	25661<br>36.95%	58502<br>45.28%
**Islands**	2040<br>6.91%	7906<br>4.53%	8404<br>6.02%	3931<br>5.75%	2990<br>5.6%	7478<br>5.36%	4206<br>6.06%	7255<br>5.33%
**Open sea**	20529<br>69.52%	128255<br>73.51%	93579<br>67.06%	47339<br>69.22%	37493<br>69.81%	94858<br>68.05%	46677<br>67.21%	94858<br>69.65%
**Shelf**	1972<br>6.68%	11754<br>6.74%	11271<br>8.08%	4674<br>6.83%	3876<br>7.22%	11557<br>8.29%	5140<br>7.4%	10682<br>7.84%
**Shore**	4987<br>16.89%	26559<br>15.22%	26281<br>18.83%	12450<br>18.2%	9333<br>17.38%	25510<br>18.3%	13431<br>19.34%	23394<br>17.18%
**1st exon**	327<br>1.1%	2532<br>1.45%	1833<br>1.31%	831<br>1.22%	592<br>1.10%	1845<br>1.33%	821<br>1.18%	1698<br>1.31%
**3'UTR**	720<br>2.44%	3515<br>2.01%	3788<br>2.71%	1627<br>2.38%	1291<br>2.4%	3827<br>2.75%	1653<br>2.38%	3557<br>2.75%
**5' UTR**	2204<br>7.47%	12470<br>7.15%	10196<br>7.31%	5289<br>7.73%	4162<br>7.75%	10158<br>7.31%	5412<br>7.79%	9441<br>7.31%
**Body**	11605<br>39.33%	60098<br>34.45%	54172<br>38.82%	26605<br>38.9%	21237<br>39.54%	53949<br>38.80%	27211<br>39.18%	50397<br>39.01%
**Exon Bnd**	137<br>0.46%	793<br>0.45%	953<br>0.68%	363<br>0.53%	282<br>0.53%	999<br>0.72%	387<br>0.56%	906<br>0.70%
**IGR**	10953<br>37.12%	70854<br>40.6%	48490<br>34.75%	24423<br>35.71%	19254<br>35.85%	48599<br>34.95%	24155<br>34.78%	45163<br>34.95%
**TSS1500**	2735<br>9.27%	17995<br>10.31%	15766<br>11.30%	7327<br>10.71%	5420<br>10.09%	15518<br>11.16%	7787<br>11.21%	14154<br>10.95%
**TSS200**	827<br>2.80%	6217<br>3.56%	4337<br>3.11%	1929<br>2.82%	1471<br>2.74%	4148<br>2.98%	2028<br>2.92%	3889<br>3.01%
[redacted]								
**Islands**	1085<br>4.43%	6037<br>3.66%	4324<br>11.09%	2364<br>7.58%	1636<br>6.79%	4085<br>6.76%	2595<br>10.11%	3885<br>6.64%
**Open sea**	18099<br>73.96%	124580<br>75.48%	22652<br>58.09%	20819<br>66.77%	17054<br>70.78%	41941<br>69.43%	15951<br>62.16%	39920<br>68.24%
**Shelf**	1684<br>6.88%	11259<br>6.82%	2569<br>6.59%	1982<br>6.36%	1629<br>6.76%	4360<br>7.22%	1670<br>6.51%	4270<br>7.3%
**Shore**	3603<br>14.72%	23177<br>14.04%	9450<br>24.43	6014<br>19.29%	3774<br>15.66%	10023<br>16.59%	5444<br>21.22%	10427<br>17.82%
**1st exon**	240<br>0.98%	2343<br>1.42%	690<br>1.77%	379<br>1.22%	256<br>1.06%	897<br>1.48%	364<br>1.42%	901<br>1.47%
**3'UTR**	616<br>2.52%	3279<br>1.99%	1010<br>2.59%	703<br>2.25%	521<br>2.16%	1387<br>2.3%	592<br>2.31%	1225<br>2%
**5' UTR**	1796<br>7.34%	11759<br>7.12%	2895<br>7.42%	2454<br>7.87%	1597<br>6.63%	4007<br>6.63%	1923<br>7.49%	3885<br>6.35%
**Body**	9774<br>39.74%	56614<br>34.3%	14144<br>36.27%	12044<br>38.63%	9276<br>38.50%	21007<br>34.77%	9849<br>38.38%	19806<br>32.36%
**Exon Bnd**	118<br>0.48%	766<br>0.46%	196<br>0.5%	132<br>0.42%	108<br>0.45%	310<br>0.51%	105<br>0.41%	291<br>0.48%
**IGR**	9927<br>37.71%	67894<br>41.13%	13170<br>37.71%	11272<br>36.15%	9552<br>39.65%	24172<br>40.01%	9201<br>35.86%	26305<br>42.98%
**TSS1500**	2087<br>8.53%	16643<br>10.08%	5253<br>13.47%	3243<br>10.4%	2146<br>8.91%	6549<br>10.84%	2713<br>10.57%	26305<br>42.98%
**TSS200**	613<br>2.51%	5755<br>3.49%	1637<br>4.2%	952<br>3.05%	637<br>2.64%	2080<br>3.44%	913<br>3.56%	2053<br>3.35%

[redacted]								
[redacted]								
**Islands**	955<br>18.88%	1869<br>19.95%	4079<br>4.06%	1567<br>4.21%	1371<br>4.63%	3393<br>4.32%	1610<br>3.68%	3370<br>4.77%
**Open sea**	2430<br>48.05%	3671<br>38.99%	70924<br>70.55%	26519<br>71.26%	20439<br>69.01%	52914<br>67.29%	30726<br>70.16%	47953<br>67.82%
**Shelf**	288<br>5.7%	495<br>5.56%	8702<br>8.66%	2692<br>7.23%	2247<br>7.59%	7197<br>9.15%	3470<br>7.92%	6412<br>9.07%
**Shore**	1384<br>27.37%	3380<br>35.90%	16830<br>16.74%	6436<br>17.29%	5559<br>18.77%	15126<br>19.24%	7987<br>18.24%	12967<br>18.34%
**1st exon**	87<br>1.72%	189<br>2.01%	1143<br>1.14%	452<br>1.21%	336<br>1.13%	948<br>1.21%	457<br>1.04%	797<br>1.13%
**3'UTR**	124<br>2.45%	236<br>2.51%	2778<br>2.76%	924<br>2.48%	770<br>2.6%	2440<br>3.1%	1061<br>2.42%	2332<br>3.3%
**5' UTR**	408<br>8.07%	711<br>7.55%	7301<br>7.26%	2835<br>7.62%	2565<br>8.66%	6151<br>7.82%	3489<br>7.97%	5556<br>7.86%
**Body**	1831<br>36.21%	3484<br>37%	40028<br>39.81%	14561<br>39.13%	11961<br>40.39%	32942<br>41.89%	17362<br>39.65	30590<br>43.27%
**Exon Bnd**	19<br>0.38%	27<br>0.29%	757<br>0.75%	231<br>0.62%	174<br>0.59%	689<br>0.88%	282<br>0.64%	615<br>0.87%
**IGR**	1726<br>34.13%	2956<br>31.4%	35318<br>35.13%	13150<br>35.34%	9702<br>32.76	24424<br>31.06%	14954<br>34.15%	21558<br>30.49%
**TSS1500**	648<br>12.81%	1350<br>14.34%	10511<br>10.46%	4084<br>10.97%	3274<br>11.05%	8968<br>11.41%	5074<br>11.59%	7418<br>10.48%
**TSS200**	214<br>4.23%	462<br>4.91%	2699<br>2.68%	977<br>2.63%	834<br>2.82%	2068<br>2.63%	1114<br>2.54%	1836<br>2.6%

Table S7. ChromHMM features of all DMRs with an FDR adjusted pvalue < 0.05 using NHEK cell annotations built from CTCF, H3K4me1, H3K4me2, H3K4me3, H3K27ac, H3K9ac, H3K36me3, H4K20me1, H3K27me3 data and plotted using R package ggplot2 (v3.3.3).

[redacted]						
**Active Promoter**	17<br>3.12%	4<br>4%	4<br>4.76%	1<br>1.02%	2<br>3.57%	2<br>1.80%
**Weak Promoter**	34<br>6.24%	8<br>8%	4<br>4.76%	3<br>3.06%	3<br>5.36%	6<br>5.41%
**Poised promoter**	24<br>4.4%	8<br>8%	9<br>10.71%	9<br>9.18%	11<br>19.64%	8<br>7.21%
**Strong enhancer**	57<br>10.46%	7<br>7%	8<br>9.52%	3<br>3.06%	3<br>5.36%	9<br>8.11%
**Weak enhancer**	83<br>15.23%	16<br>16%	15<br>17.96%	10<br>10.2%	6<br>7.14%	12<br>10.81%
**Insulator**	19<br>3.49%	5<br>5%	6<br>7.14%	6<br>6.12%	4<br>7.14%	6<br>5.41%
**Txn transition**	9<br>1.65%	1<br>1%	0<br>0%	0<br>0%	1<br>1.79%	1<br>0.9%
**Txn Elongation**	22<br>4.04%	7<br>7%	2<br>2.38%	2<br>2.04%	1<br>1.79%	4<br>3.6%
**Weak Txn**	44<br>8.07%	10<br>10%	8<br>9.52%	7<br>7.14%	3<br>5.36%	7<br>6.31%
**Repressed**	108<br>19.83%	22<br>22%	19<br>22.62%	31<br>31.63%	17<br>30.36%	34<br>30.63%
**Heterochromatin**	119<br>21.83%	12<br>12%	9<br>10.63%	26<br>26.53%	5<br>8.93%	22<br>19.82%
**Repetitive/CNV**	9<br>1.65%	0<br>0%	0<br>0%	0<br>0%	0<br>0%	0<br>0%
[redacted]						
**Active Promoter**	5<br>7.69%	8<br>2.9%	4<br>3.6%	5<br>2.81%	4<br>3.74%	6<br>3.97%
**Weak Promoter**	6<br>9.23%	16<br>5.8%	10<br>9.01%	13<br>7.3%	10<br>9.35%	13<br>8.61%
**Poised promoter**	9<br>13.85%	14<br>5.07%	3<br>2.7%	4<br>2.25%	2<br>1.87%	4<br>2.65%
**Strong enhancer**	8<br>12.31%	33<br>11.96%	20<br>18.02%	24<br>13.48%	20<br>18.69%	15<br>9.93%
**Weak enhancer**	11<br>16.92%	51<br>18.48%	24<br>21.62%	43<br>24.16%	31<br>28.97%	40<br>26.49%
**Insulator**	2<br>3.08%	7<br>2.54%	4<br>3.6%	7<br>3.93%	2<br>1.87%	5<br>3.31%
**Txn transition**	2<br>3.08%	6<br>2.17%	3<br>2.7%	1<br>0.56%	1<br>0.93%	3<br>1.99%
**Txn Elongation**	1<br>1.54%	17<br>6.16%	4<br>3.6%	12<br>6.74%	4<br>3.74%	11<br>7.28%
**Weak Txn**	3<br>4.62%	30<br>10.87%	14<br>12.61%	31<br>17.42%	13<br>12.15%	22<br>14.57%
**Repressed**	10<br>15.38%	40<br>14.49%	10<br>9.01%	19<br>10.67%	6<br>5.61%	15<br>9.93%
**Heterochromatin**	8	54	15	19	14	17

	12.31%	19.59%	13.51%	10.67%	13.08%	11.26%

Table S8. ChromHMM features of DMRs in common between BAMS and FSHD2 cells selected using an FDR adjusted pvalue < 0.05 according to NHEK cell annotations.

[redacted]		
[redacted]		
	BAMS	FSHD2
**Active Promoter**	4 (4.65%)	4 (7.41%)
**Weak Promoter**	8 (9.3%)	7 (12.96%)
**Poised promoter**	5 (5.81%)	4 (7.41%)
**Strong enhancer**	9 (10.47%)	3 (5.56%)
**Weak enhancer**	13 (15.12%)	10 (18.52%)
**Insulator**	6 (6.98%)	3 (5.56%)
**Txn transition**	1 (1.16%)	0
**Txn Elongation**	5 (5.81%)	2 (3.7%)
**Weak Txn**	11 (12.79%)	6 (11.11%)
**Repressed**	13 (15.12%)	8 (14.81%)
**Heterochromatin**	11 (12.79%)	7 (12.96%)
**Repetitive/CNV**	0	0
[redacted]		
**Active Promoter**	0	1 (11.11%)
**Weak Promoter**	0	1 (11.11%)
**Poised promoter**	2 (16.67%)	2 (22.22%)
**Strong enhancer**	2 (16.67%)	0
**Weak enhancer**	1 (8.33%)	1 (11.11%)
**Insulator**	1 (8.33%)	1 (11.11%)
**Txn transition**	0	0
**Txn Elongation**	0	0
**Weak Txn**	0	0
**Repressed**	5 (41.67%)	2 (22.22%)
**Heterochromatin**	1 (8.33%)	1 (11.11%)
**Repetitive/CNV**	0	0
[redacted]		
**Active Promoter**	2 (16.67%)	3 (10.71%)
**Weak Promoter**	2 (16.67%)	4 (14.29%)
**Poised promoter**	1 (8.33%)	1 (3.57%)
**Strong enhancer**	1 (8.33%)	2 (7.14%)
**Weak enhancer**	2 (16.67%)	5 (17.86%)
**Insulator**	0	0
**Txn transition**	1 (8.33%)	2 (7.14%)
**Txn Elongation**	0	0
**Weak Txn**	0	5 (17.86%)
**Repressed**	1 (8.33%)	3 (10.71%)
**Heterochromatin**	2 (16.67%)	3 (10.71%)
**Repetitive/CNV**	0	0
[redacted]		
**Active Promoter**	2 (3.23%)	0
**Weak Promoter**	6 (9.68%)	2 (11.76%)
**Poised promoter**	2 (3.23%)	1 (5.88%)
**Strong enhancer**	6 (9.68%)	1 (5.88%)
**Weak enhancer**	10 (16.13%)	4 (23.53%)
**Insulator**	5 (8.06%)	2 (11.76%)
**Txn transition**	1 (1.61%)	0

**Txn Elongation**	4 (6.45%)	0
**Weak Txn**	11 (17.74%)	1 (5.88%)
**Repressed**	7 (11.29%)	3 (17.65%)
**Heterochromatin**	8 (12.9%)	3 (17.65%)
**Repetitive/CNV**	0	0

Table S9. Distribution of DMPs in hiPSCs.

**HypoM**	986 (86.19%)	818 (31.33%)
**HyperM**	158 (13.81%)	1793 (68.67%)
**Islands**	672 (60.92%)	451 (17.28%)
**Open sea**	143 (12.96%)	1414 (54.18%)
**Shelf**	19 (1.72%)	168 (6.44%)
**Shore**	269 (24.39%)	577 (22.11%)
**1st exon**	103 (9.01%)	72 (2.76%)
**3'UTR**	20 (1.75%)	49 (1.88%)
**5' UTR**	55 (4.81%)	176 (6.74%)
**Body**	272 (23.8%)	865 (33.14%)
**Exon Bnd**	0	9 (0.34%)
**IGR**	370 (32.37%)	911 (34.90%)
**TSS1500**	134 (11.72%)	329 (12.61%)
**TSS2000**	189 (16.54%)	199 (7.62%)
**Islands**	15 (6.98%)	139 (5.32%)
**Open sea**	81 (51.27%)	1131 (63.08%)
**Shelf**	9 (5.7%)	132 (7.36%)
**Shore**	53 (33.54%)	391 (21.81%)
**1st exon**	3 (1.9%)	34 (1.9%)
**3'UTR**	2 (1.27%)	29 (1.62%)
**5' UTR**	12 (7.59%)	127 (7.08%)
**Body**	40 (25.32%)	621 (34.63%)
**Exon Bnd**	0	8 (0.45%)
**IGR**	52 (32.91%)	660 (36.81%)
**TSS1500**	38 (24.05%)	211 (11.77%)
**TSS200**	11 (6.96%)	103 (5.74%)
**Islands**	657 (66.7%)	312 (38.19%)
**Open sea**	102 (10.36%)	283 (34.64%)
**Shelf**	10 (1.02%)	36 (4.41%)
**Shore**	216 (21.93%)	186 (22.77%)
**1st exon**	100 (10.15%)	38 (4.65%)
**3'UTR**	18 (1.83%)	20 (2.45%)
**5' UTR**	43 (4.37%)	49 (6%)
**Body**	232 (23.55%)	244 (29.87%)
**Exon Bnd**	0	1 (0.12%)
**IGR**	318 (32.28%)	251 (30.72%)
**TSS1500**	96 (9.75%)	118 (14.44%)
**TSS200**	178 (18.07%)	96 (11.75%)

Legend to the supplementary figures

Figure S1. DNA methylation profile in control, BAMS and FSHD2 primary fibroblasts. A. Violin plot showing the Log ratio of methylation percentage (M-values) used to derive Beta values and distribution of DNA methylation levels between samples**. B.** Density plots for normalized DNA methylation levels (Beta values) in control, BAMS and FSHD2 fibroblasts showing a bimodal distribution with peaks of unmethylated and methylated sites. A significant number of sites exhibit intermediate levels of DNA methylation. **C.** Density plots for DNA methylation levels (Beta values) after batch adjustment. **D.** Hierarchical clustering of FSHD2, BAMS and control subjects for CpGs selected among the 1000 most variable position on the basis of Beta values after stringent quality control, data normalization, and removal of probes mapping to sex chromosomes. Probes associated to the same gene were aggregated by mean. **E.** 100% stacked bar graphs of the distribution of hypo- and hypermethylated probes in BAMS and FSHD2 fibroblasts. **F.** 100% stacked bar graphs for DMP by CpG content relative to CpG islands, shores (2 kb flanking CpG islands), shelves (2 kb extending from shores) or open seas (isolated CpG in the rest of the genome). **G.** 100% stacked bar graphs for DMP by features corresponding to genes first exon, 3' UTR, 5'UTR, gene bodies, Exon boundaries, Internal genomic regions (IGR), probes located 1500 bp from transcription start sites (TSS1500) or 2000 bp from transcription start sites (TSS200), in individual BAMS samples

Figure S2. Chromosomal distribution of DMPs. A. Distribution of hyperM or **B**. hypoM DMPs relative to autosomes for FSHD2 (red) or BAMS (cyan) primary fibroblasts. A filter of absolute(deltaBeta) > 0.5 was applied to the DMPs for inclusion in the chart.

Figure S3. DNA methylation profile of chromosome 6 in FSHD2 and BAMS fibroblasts. A. Distribution of DMPs in chromosome represented as a density measured in bins (bin size chr6 = 1 000 000 bp). Upper graphs correspond to hyperM DMP, lower graph to hypoM DMPs in FSHD2-14586, FSHD2-11440, FSHD2-11490, BAMS1; 2 and 9 primary fibroblasts.

Differential methylation is determined in comparison to healthy controls. **B.** Distribution of hyperM or **C**. hypoM DMRs relative to autosomes for FSHD2 (red) or BAMS (cyan) primary fibroblasts. **D**. Distribution of hyperM or **E**. hypoM DMRs relative to autosomes for FSHD2 (red) or BAMS (cyan) primary fibroblasts.

Figure S4. DNA methylation profile in control, BAMS and FSHD2 induced pluripotent stem cells. A. Violin plot showing the Log ratio of methylation percentage (M-values) used to derive Beta values and distribution of DNA methylation levels between hiPSC samples**. B.** Density plots for normalized DNA methylation levels (Beta values) in control, BAMS and FSHD2 hiPSCs showing a bimodal distribution with peaks of unmethylated and unmethylated sites. **C.** 100% Stacked Bar Graph of the distribution of hypo- and hypermethylated probes in BAMS and FSHD2 induced pluripotent stem cells. **D.** 100% Stacked Bar Graph for DMP by CpG content relative to CpG islands, shores (2 kb flanking CpG islands), shelves (2 kb extending from shores) or open seas (isolated CpG in the rest of the genome) in BAMS and FSHD2 hiPSCs. **E.** Cumulative histograms for DMP by features corresponding to genes first exon, 3' UTR, 5'UTR, gene bodies, Exon boundaries, Internal genomic regions (IGR), probes located 1500 bp from transcription start sites (TSS1500) or 2000 bp from transcription start sites (TSS200), in BAMS and FSHD2 hiPSCs. Values are presented in supplementary table 3. **F.** Venn Diagram for DMPs in BAMS and FSHD2 hiPSCs.

Figure S5. Gene expression profiling in fibroblasts and induced pluripotent cells from patients carrying a mutation in *SMCHD1*. A. Heatmap of RNAseq data (TPM values with a row sum >1, distance: Manhattan, Clustering: Ward.D2) for gene expressed in primary fibroblasts from healthy donors (controls, CT), patients affected with BAMS or FSHD2. **B.** Volcano plots for genes differentially regulated in BAMS (upper panel) or FSHD2 (lower panel) cells versus controls. Fold changes (FC log 2) are compared to the number of reads (logCounts, 58219 variables). Black dots represent genes that did not reach significance whereas dysregulated genes are shown in red. **C.** Heatmap of RNAseq data (TPM values,

distance: Manhattan, Clustering: Ward.D2) for gene expressed in induced pluripotent stem cells from healthy donors (controls, CT), patients affected with BAMS or FSHD2. **D.** Volcano plots for genes differentially regulated in BAMS (upper panel) or FSHD2 (lower panel) hiPSCs versus controls. Fold changes (FC log 2) are compared to the number of reads (logCounts, 58219 variables). Black dots represent genes that did not reach significance whereas dysregulated genes are shown in red. **E.** Venn diagrams for DEGs in primary fibroblasts for the different categories of patients compared to controls and intersection with the list of DUX target genes (7). DUX4 target genes that are differentially expressed in the different categories are indicated with 6 genes in FSHD2, 14 in BAMS and 5 common to the two diseases. **F**. Venn diagrams for DEGs in hiPSCs for the different categories of patients compared to controls and intersection with the list of DUX target genes (7). Only one gene, *OAS1* is differentially expressed in BAMS cells.

Figure S6. Germline *SMCHD1* mutations result in changes in *HOX* genes expression and methylation profiles. A. Hierarchical clustering of differential methylation at *HOXA* gene loci (chr7: 27,064,176-27,227,600) in BAMS, FSHD2 and Controls with Ward's method on Euclidian distance for the different samples. **B**. Hierarchical clustering of differential methylation at *HOXC* gene loci in BAMS, FSHD2 and Controls with Ward's method on Euclidian distance for the different samples. **C**. Representative distribution of the DNA methylation profile of DMPs at the *HOXA* locus in the different FSHD2 (11440, 11491, 14586, red) or BAMS (BAMS1, BAMS2, BAMS9) samples. Black curves correspond to HyperM probes, white curves to HypoM ones. **D**. Representative distribution of the DNA methylation profile of DMPs at the *HOXC* locus (chr12: 53,908,560-54,069,307) in the different FSHD2 (11440, 11491, 14586, red) or BAMS (BAMS1, BAMS2, BAMS9) samples. **E.** Venn diagrams for comparison of *HOX* genes that are differentially expressed in FSHD2 and BAMS fibroblasts compared to controls with a fold-change >2 and <-2 and an FDR <0.05. **F.** Table of differentially expressed *HOX* genes in FSHD2 and BAMS cells. Red dots correspond to genes that are upregulated and green dots, to downregulated ones. Fold Change (FC) is indicated.

Figure S7. DNA methylation profiling of *HOXB* and *D* genes in SMCHD1-deficient primary fibroblasts.

A. Representative distribution of the DNA methylation profile of DMPs at the *HOXB* locus in the different FSHD2 (11440, 11491, 14586, red) or BAMS (BAMS1, BAMS2, BAMS9, Cyan) samples. **B**. Hierarchical clustering of FSHD2, BAMS and control subjects based on Beta values of aggregated probes (mean) to *HOXB* genes (chr17: 48,496,690-48,772,940, distance: Canberra, clustering Ward D2). **C**. Representative distribution of the DNA methylation profile of DMPs at the *HOXD* locus in the different FSHD2 (11440, 11491, 14586, red) or BAMS (BAMS1, BAMS2, BAMS9, Cyan) samples. **D**. Hierarchical clustering of FSHD2, BAMS and control subjects based on Beta values of aggregated probes (mean) to HOXD genes (chr2: 176,069,968-176,201,505 (8-10), distance: Canberra, clustering Ward D2).

Figure S8. Bisulfite sequencing for DNA methylation profiling of *HOX* genes. DNA methylation was determined after sodium bisulfite modification for 4 *HOX* genes DMRs for primary fibroblasts (left panels) or corresponding hiPSC clones (right panels) for *HOXA13* (**A**), *HOXB2* (**B**), *HOXB6* (**C**) *or HOXC4/5/6* (**D**). For each sequence, cumulative histogram bars represent the percentage of methylated CpG at each position of the sequence analyzed (black) and the percentage of unmethylated CGs (white bars).

Figure S9. Analysis of *HOX* genes in wild-type HEK and HEK *SMCHD1*-KO cells.

A. D4Z4 methylation level was evaluated in control primary fibroblasts, after *SMCHD1* knock-down following transient transduction of lentiviral particles producing ShRNA targeting the *SMCHD1* transcript or a scramble ShRNA as control. DNA methylation of the DR1 region was analyzed as described (5) and reported for the different CpG (black, percentage of methylated CGs, white, percentage of unmethylated CG). The global methylation level is indicated for each sample. SMCHD1 KD was validated by RT-qPCR. Comparing the results of our Scramble (Scr) and SMCHD1-inhibited cells (Sh) to our original untreated cells (Ag08), the relative level

of SMCHD1 expression was respectively 106.27% in cells transfected with the scramble ShRNA and 47.42%, in *SMCHD1*-KD cells. *SMCHD1*-KD was also validated by western blotting (not show). **B.** We analyzed SMCHD1 binding to the different HOX DMRs (*HOXA13*, *HOXB2*, *HOXB6* and *HOXC4/5/6) in* HEK *and* HEK-*SMCHD1*-KO cells. Enrichment over input was determined by qPCR. Bar plots display the average enrichment after normalization over a single copy intergenic region. Values are average from at least three independent biological replicates and a technical duplicate for each. Error bar represents standard error. Statistical significance was determined using a paired two-tailed student's t test (* $p< 0.01$; ** $p< 0.001$). **C.** Cumulative histogram display DNA methylation profiles analyzed in HEK cells and cells in which *SMCHD1* gene expression was invalidated by gene editing (HEK KO) for *HOXA13*, *HOXB2*, *HOXB6* and *HOXC4/5/6* DMRs. Black bars correspond to the percentage of methylated sites, white bars to unmethylated sites. **D.** Expression of different *HOX* genes was evaluated by RT-qPCR performed as biological and technical in triplicates in HEK and HEK KO cells. Expression was normalized to three housekeeping genes (*GAPDH*, *HPRT* and *PPIA*). Statistical significance was determined by Kruskal-Wallis statistical test. ** p-value<0.005, **** p-value<0.00005.

Figure S10. Somatic loss of heterozygosity at the 18p11.32 locus encompassing the *SMCHD1* gene is not associated with D4Z4 hypomethylation in breast cancer tumors. Graphs display the methylation rate (*y-axis*) related to the log2 ratio (*x-axis*) of 18p loss in 29 breast cancer tumors with a loss of 18p heterozygosity encompassing the *SMCHD1* gene locus as determined by comparative genomic hybridization (CGH). The correlation value and level of significance is indicated for the different samples.

Figure S11. Chromatin profiling in BAMS and FSHD2 muscle cells.

A. Distribution of CTCF, H3K27me3, H3K27Ac and H3K4me3 relative to the different autosomes in Controls (CT2, black bars), BAMS (cyan bars) or FSHD2 (red bars) muscle

fibers. **B**. Distribution of peaks width for CTCF, H3K27me3, H3K27Ac or H3K4me3 in Control BAMS1 and FSHD2 Neural Crest Stem Cells.

Figure S12. Chromatin profiling of hiPSC-derived muscle fibers and neural crest stem cells from SMCHD1-deficient patients. A. Distribution of distances from Transcription Start Sites (TSS) for peaks enriched in H3K27Ac or H3K4me3 in Controls (CT), BAMS or FSHD2 muscle fibers (MF) or neural crest stem cells (NCSCs). Peaks distribution relative to TSS was assessed using the chipenrich (v2.14.0) R package. **B.** Distribution of peaks enriched in H3K4me3 relative to chromatin features determined using the ChromHMM track in MF and NCSC derived from Control, BAMS or FSHD2 hiPSCs. Peaks with at least a qvalue < 0.05 in one replicate were analyzed for chromHMM features using HSMM (Human Skeletal Muscle Myoblasts) cells annotations and represented as barplot using the R ggplot2 (v3.3.3) package.

Figure S13. For a number of sites, loss of SMCHD1 binding leads to increased CTCF enrichment.

Relative quantification of SMCHD1 or CTCF enrichment after ChIP-qPCR in HEK293 cells and HEK293 KO cells for putative SMCHD1 sites selected from publicly available ChIP-Seq data (https://www.ncbi.nlm.nih.gov/geo/query/acc.cgi?acc=GSM1130654) at the *NCAM2*, *BET1L*, *SEMA5A* and *WASH7P* loci. The different proteins targeted by immunoprecipitation are indicated on the *x-axis* (SMCHD1, CTCF). Each experiment was done in two biological replicates with each biological replicate analyzed in technical duplicates. CT values were normalized to the input. Fold change was calculated and values were normalized to an intergenic unique sequence on chromosome 5. Statistical significance was determined using a 2way ANOVA test for multiple comparisons (* p value < 0.1; **** p value < 0.001).

Figure S14. D4Z4 subfragments harbor SMCHD1-dependent *cis*-regulatory activity

A. Schematic representation of the D4Z4 element from position 1 to 3303 given relative to the two flanking *Kpn*I sites (K) (to scale). The different regions within *D4Z4* are indicated: *LSau*

repeat (position 1-340), Region A (position 869-1071), *hhspm3* (position 1313-1780), *DUX4* ORF (position 1792-3303). Different fragments in the proximal part of the repeat encompassing to the most differentially methylated regions (DR1, position 566-819 and 5P, position 1027-1253 relative to the first *Kpn*I site) were cloned in the pGL3 basic vector lacking the SV40 promoter and enhancer, the pGL3 Enhancer vector lacking the SV40 promoter or the pGL3 Promoter vector lacking the SV40 enhancer. Expression of the firefly luciferase was determined 48 hrs post-transfection in HEK cells or HEK KO cells. Firely luciferase levels were normalized to expression of the Renilla luciferase used as transfection control. Values corresponding to the luciferase activity (expressed in arbitrary units, A.U.) are the average of at least three independent assays with three measures per experiment (n=9). Error bar represents standard error. Statistical significance was determined using a Mann-Whitney test, * p value = 0.1; ** p value <0.01, *** p value < 0.001; **** p value < 0.0001. **B.** Relative luminescence activity (RLU) for the different constructs indicated on the left of the histogram after transfection in HEK (black bars) or HEK-KO cells (grey bars). **C**. Different subfragments corresponding to the DR1 fragment were cloned in the pGL3 promoter vector. This sequence contains 31 CpG sites that are differentially methylated in cells from patients with a mutation in SMCHD1 (BAMS and FSHD). Fragments containing CG1-10; 10-20 or 21-30 were tested together with fragments lacking these different elements (ΔCG1-20; ΔCG10-20; ΔCG21-31). **D**. Different sequences to be tested were cloned downstream of the *eGFP* reporter gene: *HOXB2* DMR, *HOXB6* DMR, *SEMA5A*, *NCAM2*, *BET1L, WASH7PL*. Linearized plasmids were transfected into cells. Stable eGFP expression was measured by flow cytometry (FACS) for an extended period of time in cells grown in the presence of Hygromycin B. Representative spectra of the % of eGFP positive cells a presented. eGFP expression level is the average of 5 measurements from day 18 to day 40 post-transfection, when *eGFP* expression reaches a plateau, of three independent assays ± S.D. For each condition, eGFP expression was compared to values obtained in cells transfected with the empty vector (pCMV). In HEK KO cells, eGFP expression was also measured 72 hrs after transfection of a SMCHD1 expression

vector (grey curves). Asteriks indicate statistically significant values relative to control vectors (pCMV) (Student's t test). * $p < 0.001$; ** $p < 0.005$; *** $p < 0.05$.

Figure S15. Biological pathways associated to H3K4me3 or H3K27Ac peaks in BAMS or FSHD2 hiPSC-derived muscle fibers and neural crest stem cells.

A. Venn diagrams for comparison of peaks enriched in Controls, BAMS and FSHD2 hiPSC-derived muscle fibers (MF) for H3K4me3 or H3K27Ac. **B**. GO terms for BP corresponding to peaks enriched in H3K4me3 or H3K27Ac. Light grey bars in the right represent the enrichment score (Log10 of False Discovery Rate) for each GO-term. Cyan bars correspond to the number of genes corresponding to the different BP in BAMS MF, red bars to BP enriched in FSHD2 cells, **C**. Venn diagrams for comparison of peaks enriched in Controls, BAMS and FSHD2 hiPSC-derived neural crest stem cells (NCSC) for H3K4me3 or H3K27Ac. **D**. GO terms for BP corresponding to peaks enriched in H3K4me3 or H3K27Ac. Light grey bars in the right represent the enrichment score (Log10 of False Discovery Rate) for each GO-term. Cyan bars correspond to the number of genes corresponding to the different BP in BAMS NCSCs, red bars to BP enriched in FSHD2 NCSCs,

Supplementary references

1. Gordon, C.T., Xue, S., Yigit, G., Filali, H., Chen, K., Rosin, N., Yoshiura, K.I., Oufadem, M., Beck, T.J., McGowan, R. *et al.* (2017) De novo mutations in SMCHD1 cause Bosma arhinia microphthalmia syndrome and abrogate nasal development. *Nat Genet*, **49**, 249-255.
2. Dion, C., Roche, S., Laberthonniere, C., Broucqsault, N., Mariot, V., Xue, S., Gurzau, A.D., Nowak, A., Gordon, C.T., Gaillard, M.C. *et al.* (2019) SMCHD1 is involved in de novo methylation of the DUX4-encoding D4Z4 macrosatellite. *Nucleic Acids Res*, **47**, 2822-2839.
3. Badja, C., Maleeva, G., El-Yazidi, C., Barruet, E., Lasserre, M., Tropel, P., Binetruy, B., Bregestovski, P. and Magdinier, F. (2014) Efficient and cost-effective generation of mature neurons from human induced pluripotent stem cells. *Stem Cells Transl Med*, **3**, 1467-1472.
4. Snider, L., Geng, L.N., Lemmers, R.J., Kyba, M., Ware, C.B., Nelson, A.M., Tawil, R., Filippova, G.N., van der Maarel, S.M., Tapscott, S.J. *et al.* (2010) Facioscapulohumeral dystrophy: incomplete suppression of a retrotransposed gene. *PLoS Genet*, **6**, e1001181.
5. Roche, S., Dion, C., Broucqsault, N., Laberthonniere, C., Gaillard, M.C., Robin, J.D., Lagarde, A., Puppo, F., Vovan, C., Chaix, C. *et al.* (2019) Methylation hotspots evidenced by deep sequencing in patients with facioscapulohumeral dystrophy and mosaicism. *Neurol Genet*, **5**, e372.
6. Gaillard, M.C., Roche, S., Dion, C., Tasmadjian, A., Bouget, G., Salort-Campana, E., Vovan, C., Chaix, C., Broucqsault, N., Morere, J. *et al.* (2014) Differential DNA methylation of the D4Z4 repeat in patients with FSHD and asymptomatic carriers. *Neurology*.
7. Geng, L.N., Yao, Z., Snider, L., Fong, A.P., Cech, J.N., Young, J.M., van der Maarel, S.M., Ruzzo, W.L., Gentleman, R.C., Tawil, R. *et al.* (2012) DUX4 activates germline genes, retroelements, and immune mediators: implications for facioscapulohumeral dystrophy. *Dev Cell*, **22**, 38-51.
8. Shaw, N.D., Brand, H., Kupchinsky, Z.A., Bengani, H., Plummer, L., Jones, T.I., Erdin, S., Williamson, K.A., Rainger, J., Stortchevoi, A. *et al.* (2017) SMCHD1 mutations associated with a rare muscular dystrophy can also cause isolated arhinia and Bosma arhinia microphthalmia syndrome. *Nat Genet*, **49**, 238-248.
9. Lemmers, R.J., Tawil, R., Petek, L.M., Balog, J., Block, G.J., Santen, G.W., Amell, A.M., van der Vliet, P.J., Almomani, R., Straasheijm, K.R. *et al.* (2012) Digenic inheritance of an SMCHD1 mutation and an FSHD-permissive D4Z4 allele causes facioscapulohumeral muscular dystrophy type 2. *Nat Genet*, **44**, 1370-1374.
10. Gordon, C.T., Xue, S., Yigit, G., Filali, H., Chen, K., Rosin, N., Yoshiura, K.I., Oufadem, M., Beck, T.J., McGowan, R. *et al.* (2017) De novo mutations in SMCHD1 cause Bosma arhinia microphthalmia syndrome and abrogate nasal development. *Nat Genet*, **49**, 249-255.
