# Supplementary material for: In skeletal muscle and neural crest cells, SMCHD1 regulates biological pathways relevant for Bosma syndrome and facioscapulohumeral dystrophy phenotype
Source: Nucleic Acids Res. 2023 Jun 19;51(14):7269–87. doi: 10.1093/nar/gkad523 (PMC10415154; doi:10.1093/nar/gkad523)
Supplement: gkad523_Supplemental_Files [file gkad523_supplemental_files.zip › List of supplementary files.docx]

**List of supplementary files.**

**File S1,** Differentially methylated probes (DMPs) and regions (DMRs) in fibroblasts and hiPSCs

**File S2**, Genes that are differentially expressed (DEGs) in fibroblasts and hiPSCs

**File S3**, list of CTCF, H3K4me3, H3K27Ac and H3K27me3 peaks in control, BAMS and FSHD2 muscle fibers (MF).

**File S4,** list of CTCF, H3K4me3, H3K27Ac and H3K27me3 peaks in control, BAMS and FSHD2 neural crest stem cells (NCSCs).

**File S5,** overlap between muscle fibers or neural crest cells DEGs and genes associated with a CTCF or H3K27me3 peak
